# Supplementary material for: Bone microstructure and the evolution of growth patterns in Permo-Triassic therocephalians (Amniota, Therapsida) of South Africa
Source: PeerJ. 2014 Apr 8;2:e325. doi: 10.7717/peerj.325 (PMC3994631; doi:10.7717/peerj.325)
Supplement: Supplemental Information 1 [file peerj-02-325-s001.docx]

**Supporting Data for Huttenlocker & Botha-Brink**

**‘Bone microstructure and the evolution of growth patterns in Permo-Triassic therocephalians (Amniota, Therapsida) of South Africa’**

## Contents:

Detailed Description…………………………………………………………………………….S-2

References……………………………………………………………………………………..S-30

Supporting Figures (Captions)………………………………………………………………...S-32

## Detailed Description

THEROCEPHALIA

Lycosuchidae

*Lycosuchus vanderrieti*

(Fig. S1)

**General—**SAM-PK-9084 (radius, ulna), from Reitkuil, Beaufort West (Middle Permian, *Tapinocephalus* AZ); SAM-PK-K9012 (femur), from Klein Koedoes Kop, Beaufort West (Middle Permian, *Tapinocephalus* AZ). The histological profiles of long bone shafts reveal well-vascularized cortical bone with multiple growth zones incorporating nonlamellar interstitial ECM (including woven- and parallel-fibered tissue) with reticular to subplexiform primary osteons, interrupted by cyclic growth marks (annuli or LAGs). Secondary osteons are apparently absent in all elements.

**Radius—**The radius (Fig. S1A, B) is a robust element with a circular cross-section at mid-diaphysis, revealing a compact cortex that grades into a porous spongiosa (the medullary region largely being occluded by cancellous bone). The bone wall thickness at the midshaft is moderate (*RBT*, 16%; *K*, 0.42) and composed of FLB with many large primary osteons. The osteons are arranged into both longitudinal and reticular orientations. The interstitial ECM is woven-fibered, but with some parallel-fibered tissue deposited periodically near growth marks. Intervening osteocyte lacunae are highly concentrated and disorganized between adjacent primary osteons and are generally globular in shape (resembling the ‘static osteogenic-derived woven bone’ of Stein and Prondvai, 2013). The densely packed longitudinal and reticular primary osteons are distributed in an ordered fashion within circularly organized growth zones. The canals become smaller and more sparsely distributed in the outermost cortex where they are also predominantly longitudinal in orientation. The average mid-cortical vascularity (%*CV*) is 10.7 and the mean primary osteon diameter (*POD*) is 101 µm. Tissue structures, including growth marks, are well visible and there are at least three growth marks (LAGs) preserved in the cortex. The spacing of successive growth marks decreases toward the periosteal surface, and there is a strongly defined LAG in the outer region where the vasculature becomes more sparse and oriented longitudinally. Although apposition rate likely decreased toward the outer perimeter, there is no external fundamental system (EFS).

**Ulna—** The ulna (Fig. S1C) bears a broad, flat midshaft such that the cross-sectional profile is roughly ellipsoid to figure-8. As in the radius, the moderately porous cortex grades into a more cancellous medullary region that is largely occluded by fine cancellous bone and a few trabecular structures. The cortex is somewhat thicker than in the radius (*RBT*, 25%; *K*, 0.38). A small marrow cavity is present. The cortex consists of fibrolamellar structure, composed of primary osteons within a woven-fibered interstitial ECM but with some parallel-fibered tissue. The dense and disorganized lacunocanalicular network between primary osteons resembles that of the radius. The primary osteon orientation is predominantly reticular and radial. The %*CV* is 15.7 and the mean *POD* is 109 µm. The vascular canals (which are of moderate size) become finer and more sparsely distributed in the outermost cortex, indicative of growth attenuation. The vasculature is ordered within growth zones as in the radius, several of which can be discerned by well-defined LAGs. There are as many as four preserved LAGs in total. The LAGs become more closely spaced near the periosteal surface as in the radius. Although the outer region of the bone becomes more parallel-fibered, there is no EFS in the subperiosteal region.

**Femur—**The femur (Fig. S1D, E) is a short, robust bone with a compressed shaft that is oval in cross-section (unlike the rounded cross-section of later eutherocephalians). In section, the midshaft is of moderate thickness, but thinner than the forelimb elements (*RBT*, 15%; *K*, 0.67). It is composed of FLB with large primary osteons of varying orientations. The vascular motifs are somewhat complex, the predominant orientation being subplexiform, but with some reticular and radial osteons at muscular attachment sites, such as the adductor ridge. The degree of vascularization of the femur is generally greater than in the radius and ulna (%*CV*, 16.9; mean *POD*, 168 µm). Growth marks are difficult to distinguish due to the circular arrangement of primary osteons within the subplexiform network, although three faint bands (annuli?) of parallel-fibered bone can be observed in the posteroventral quadrant where the osteons form a more reticular arrangement. Faint bundles of Sharpey’s fibers are also evident near the adductor ridge in the outer cortex, indicating attachment of the adductor musculature. The matrix composing the outermost cortex is highly birefringent, indicative of its strong parallel-fibered and lamellar structure. The lacunocanalicular network also includes more lenticular osteocyte lacunae that are sparsely distributed. Although the bone being laid down in this region was no longer fibrolamellar and the vascular canals became sparser, the tissue lacks the avascular and multi-lamellar structure characteristic of a true EFS.

SCYLACOSAURIA

Scylacosauridae

*Glanosuchus macrops*

(Fig. S2A)

**General**—BP/1/6228 (ulna), from the Grant, Albany (Middle Permian, *Tapinocephalus* AZ).

**Ulna**—The ulna (BP/1/6228) is a relatively flat element with an ellipsoid to figure-8 cross-section along the midshaft. The bone is relatively dense with a highly cancellous internal structure deep to the cortex with poor development of the marrow cavity. The cortex is moderately thick (*RBT*, 20%; *K*, 0.42) and composed of fibrolamellar and parallel-fibered bone with primary osteons, periodically interrupted by growth marks. Localized areas contain lenticular osteocyte lacunae, particularly in the outer cortex, although the majority of osteocyte lacunae are globular and haphazardly organized. Secondary structure is limited; secondary osteons are apparently absent, but resorption cavities formed in the deep cortex near the compact-cancellous transition. The vascular network is predominantly made up of longitudinal primary osteons, which are distributed most densely in the anterior (preaxial) and posterior (postaxial) sides of the bone where the growth zones are thickest. The longitudinal primary osteons are generally largest deeper in the cortex, and become smaller and more sparsely distributed in the outer cortex, closest to the subperiosteal region. However, in the outer cortex along the anterior and posterior margins, the primary osteons generally maintain their size and form a slightly more reticular motif. The %*CV* is 4.9 and the average primary osteon diameter is 51 µm, therefore having a comparatively lesser degree of vascularization than in *Lycosuchus*. Growth zones delimited by opaque growth marks are easily distinguishable in the specimen. There are at least three annuli traceable around the bone. Their spacing does not decrease toward the periphery, and the outermost growth zones maintain moderate vascularity with reticular FLB in some areas. Primary osteons were still forming in the subperiosteal region and no EFS was laid down by the time of death.

Scylacosauridae indet.

(Fig. S2B-E)

**General—**BP/1/5576 (radius, ulna), from Combrinckskraal, Prince Albert District (Middle Permian, *Tapinocephalus* AZ); BP/1/5587 (humerus, radius, ulna), from Wolwekuil, Victoria West (Middle Permian, *Tapinocephalus* AZ); CGS R300 (humerus), locality unknown; SAM-PK-5018 (humerus, radius, femur, tibia, fibula), from Abrahamskraal, Prince Albert District (Middle Permian, *Tapinocephalus* AZ); SAM-PK-11557 (fibula), from De Cypher, Beaufort West (Middle Permian, *Tapinocephalus* AZ). Long bone microstructure has been described in scylacosaurids (Ricqlès, 1969; Ray, Botha & Chinsamy, 2004; Chinsamy-Turan, 2012), but previous genus-level identifications of these specimens are dubious due to inadequate diagnostic skull material. As two of the studied specimens have been described elsewhere (SAM-PK-5018, 11557) the present analysis serves to supplement these earlier descriptions with additional material.

**Humerus—**The humerus is a robust bone with a thick cortex at midshaft (*RBT*, ~30%; *K*, ~0.30) and an open marrow cavity with a few coarse trabeculae and lined by endosteal lamellae. The cortex is composed predominantly of FLB with densely packed primary osteons having a variety of orientations in localized regions. Ray, Botha & Chinsamy (2004) reported a laminar to subplexiform arrangement of osteons with occasional subreticular osteons in localized regions, corroborated here by study of additional specimens (BP/1/5587, CGS R300). Independent observations suggest that a subplexiform pattern of vascularization in the humerus midshaft was characteristic of scylacosaurids, but with strongly oblique or reticular patterns localized near sites of muscle insertion that are also associated with Sharpey’s fibers (i.e., deltopectoral crest). No secondary osteons are present in the humerus. The %*CV* is ~13-15% and the average primary osteon diameter bears a wide range of ~75-112 µm, which is generally high for therapsids. The interstitial ECM is more parallel-fibered than woven and the primary osteons are lined by thick circumferential lamellae, so that there is strong birefringence under crossed-polarized light. Osteocyte lacunae are well concentrated, globular and disorganized, but are more lenticular where they are associated with streaks of parallel-fibered bone preceding growth marks. Parallel-fibered bone is common in growth increments immediately preceding growth marks (LAGs), indicating decelerating growth before periodic cessation. The number of preserved growth marks varies among individuals, but ranges from three to five LAGs.

**Radius—**The radius midshaft is subtriangular in cross-section and bears a moderately thick bone wall (*RBT*, ~17-23%; *K*, ~0.46-0.47) with an open marrow cavity lined by sparse trabecular structures, but generally with a sharp delineation of the cortex. Primary osteons are scattered throughout the cortex in a predominantly longitudinal orientation, though canals are more obliquely radial in localized areas such as the attachment site of the interosseous membrane. The interstitial ECM is more parallel-fibered than woven and punctuated by occasional growth marks. Areas of parallel-fibered bone bear more sparsely distributed and ordered lenticular osteocyte lacunae, especially in the outer cortex and near growth marks. In BP/1/5587, the exact number of growth marks is difficult to discern and the outer layers of bone appear to have been actively deposited during the time of death (with primary osteons forming around the subperiosteal region). By contrast, BP/1/5576 exhibits four or more growth marks that appear in the outer half of the cortex and become progressively closer spaced toward the subperiosteal margin, indicating that growth had slowed prior to death in spite of its smaller size compared to BP/1/5587. However, there was no EFS as in the other studied elements.

**Ulna—**The histological profile of the ulna is near identical to the radius, although the oblate nature of the bone with wider growth zones allows the number of growth marks to be more reliably estimated. BP/1/5587 exhibits at least three thick growth zones with sparse longitudinal and reticular osteons and weakly radial osteons along the expanded anterior and posterior sides of the bone. These osteons were still being formed at the time of death, corroborating the observations from the radius of the same individual. BP/1/5576 differs in that as few as five (and as many as six) growth marks accompanied by incremental lamellae (annuli) are apparent in the outer cortex, becoming closer spaced toward the subperiosteal margin as in the radius. The primary osteons are longitudinally oriented with occasional subreticular orientations in some areas. Among-individual histovariation suggests the existence of variation in subadult growth rates within these two scylacosaurids.

**Femur—**The femur of SAM-PK-5018 was described by Ray, Botha & Chinsamy (2004). It bears a relatively thin bone wall compared to the forelimbs that is composed of moderately vascularized FLB. The primary osteons are predominantly longitudinal to subreticular in orientation. The perimedullary region bears extensive resorption cavities and a few secondary osteons as reported by Ray, Botha & Chinsamy (2004). There were apparently no growth marks preserved in the specimen.

**Tibia—**The tibia (SAM-PK-5018) is a thick bone, though with an open marrow cavity and moderately thick bone wall as in the forelimb epipodial elements (*RBT*, 24%; *K*, 0.60), but thinner than the humerus. The cortex is predominantly composed of FLB with subreticular primary osteons as described by Ray, Botha & Chinsamy (2004). Additionally, there are at least three growth marks visible (a faint inner annulus and two sharply defined LAGs in the outer cortex) demarcating growth zones of variable thicknesses. Toward the periphery the primary osteons become more obliquely oriented, taking on a strong radial orientation in the outer two growth zones and communicating with the periosteal surface (indicating active bone growth at the time of death). The lacunocanalicular network is too poorly preserved to be described and osteocyte lacunae are not discernible. Secondary osteons are absent.

**Fibula—**The fibula is extremely poorly preserved in SAM-PK-5018, primarily due to obliteration of the outer cortical surface, thereby making growth interpretations difficult. Deep portions of the cortex in this specimen reveal large erosion cavities and secondary osteons. Discussion of primary growth in the fibula is herein limited to SAM-PK-11557, which preserves a thick cortical wall with coarse trabeculae surrounding a marrow cavity. Resorption cavities are extensive throughout the perimedullary region and are lined endosteally by lamellar bone. The fibrolamellar cortex is densely compacted with large, infilled primary osteons tightly packed and predominantly longitudinal in orientation (though with sparse reticular osteons and anastomoses). The lacunocanalicular network between osteons is formed by haphazardly arranged globular osteocyte lacunae. The exact number of growth marks is difficult to discern, but a relatively thick growth zone with abundant primary osteons gives way to a series of closely spaced annuli near the periphery. The vasculature in this outer region also became sparse, suggesting an overall decrease in growth prior to death. Osteocyte lacunae in this region were more sparse and lenticular in shape. Secondary osteons are occasionally present in the deep cortex, diagnosed clearly by their scalloped cement lines (Fig. S2B).

EUTHEROCEPHALIA

Akidnognathidae

*Olivierosuchus parringtoni*

(Fig. S3A-C, E, F)

**General**—NMQR 3605 (humerus), from Barendskraal, Middleburg (Lower Triassic, *Lystrosaurus* AZ); SAM-PK-K10617 (femur), from Wapadsberg Pass, Zeekoegat, Graaff-Reinet District (Lower Triassic, *Lystrosaurus* AZ). The cross-sectional profiles of limb bone shafts in *Olivierosuchus* reflect general tendencies in other therocephalians, in that the forelimb was robust and thick-walled whereas the hindlimb was relatively gracile and thinner-walled (the humeral cortex being extremely thickened and marrow cavity occluded; the femur having a relatively open marrow cavity with few trabeculae). The cortex predominantly incorporates a nonlamellar interstitial ECM with dense, reticular vascularization as in *Moschorhinus* (Huttenlocker and Botha-Brink, 2013). Growth marks are few and confined to the outer cortex and secondary reconstruction is limited. There are no secondary osteons.

**Humerus**—The humerus (NMQR 3605) (Fig. S3A-C) bears a prominent deltopectoral crest that traverses most of the length of the shaft, producing a subtriangular or tear-shaped cross-section as in *Moschorhinus* and other medium-to-large therocephalians. Notably, the bone is extremely compacted in section and most of the medullary region is occluded by coarse trabeculae such that the marrow cavity is extremely reduced (approximate radius of cavity ~ 2 mm). As a result, the cortex is extremely thick (*RBT*, 36%; *K*, 0.38). The cortical bone consists of both longitudinal and reticular primary osteons in a woven-fibered interstitial ECM, although the matrix becomes more parallel-fibered in the outer half of the cortex. The large, intervening osteocyte lacunae between osteons are numerous between primary osteons, highly concentrated and almost exclusively globular in shape. The cortical bone is moderately well-vascularized and the primary osteons are large (%*CV*, 9.9; mean *POD*, 99 µm). Several of these features resemble *Moschorhinus*, including the occluded lumen, disorganized woven-fibered matrix, abundant reticular canals, and large primary osteons. Kitching (1977) suggested that *Olivierosuchus* might represent a juvenile of *Moschorhinus*. However, the specimen NMQR 3605 is interpreted here as near somatic maturity as suggested by Botha-Brink and Modesto (2011). It is the largest known specimen of its kind (BSL = 122 mm), exhibits fused neurocentral sutures, and records the transition to outer parallel-fibered bone in the humeral cortex with two distinct LAGs indicating that growth had slowed down over two seasons. This contrasts with the condition in a slightly larger *Moschorhinus* specimen from coeval Triassic rocks (SAM-PK-K118) which shows heavily vascularized FLB with no growth marks (Fig. S3C, D).

**Femur**—The femur (SAM-PK-K10617) (Fig. S3E, F) is more elliptical in cross-section, although some dorsoventral compression has distorted the shaft slightly, flattening and cracking it. The cortical bone is relatively well preserved, revealing a somewhat thinner diaphyseal bone wall than in the humerus. Some coarse cancellous bone is present around the perimedullary region. The cortex is composed of FLB with longitudinal primary osteons. Osteocyte lacunae are large, highly concentrated and haphazardly distributed. There is a moderate to weak degree of vascularization, although the osteons are relatively large (%*CV*, 3.4; mean *POD*, 75 µm). The interstitial ECM is predominantly woven-fibered but with some patches of parallel-fibered bone in the outer cortex. There are no growth marks visible in the specimen and no secondary osteons.

Hofmeyriidae

*Hofmeyria atavus*

(Fig. S4)

**General**—BP/1/4404 (humerus, radius, ulna), from Matjiesfontein (Highlands), Victoria West (Upper Permian, *Cistecephalus* AZ). The bones are remarkably robust and thick-walled relative to the size of the individual (BSL = 67 mm). The cross-sectional profile of the mid-diaphysis in all bones includes a distinct central cavity with a smooth, circular rim of endosteal lamellar bone (rather than irregularly organized cancellous bone tissue). The tissue composition of the cortex is generally more parallel-fibered than woven and the outer cortex of the epipodials preserves numerous annular growth marks. Primary osteons are primarily longitudinally oriented (though with many reticular connections in the humerus) and mostly reside in the inner half to two-thirds of the cortex. There are no secondary osteons in any of the studied elements.

**Humerus**—The humeral diaphysis (Fig. S4A) bears a relatively compact cross-sectional profile as in most of the other elements, given its thick cortex (*RBT*, 31%; *K*, 0.32) and sharp boundary between the cortical bone wall and the diminutive central cavity. There are a few coarse trabeculae around the marrow cavity, rather than the extensive cancellous tissue observed in earlier therocephalians. The interstitial ECM of the cortical bone appears to incorporate some woven-fibered structure, but is primarily parallel-fibered, especially nearing the subperiosteal margin. Osteocyte lacunae are primarily large, globular, and well-concentrated. The primary osteons are fairly densely-packed throughout much of the cortex and form a reticular network with occasional longitudinal canals. The cortex is moderately vascularized, although the primary osteons are relatively small (%*CV*, 7.5; mean *POD*, 72 µm). Growth marks are not easily distinguishable in the humerus. However, when viewed under polarized light, three bundles or ‘waves’ of highly birefringent parallel-fibered bone are visible in the outer half of the cortex, possibly indicating cyclic alternations in rate of bone accretion. Parallel-fibered bone predominates in the outer cortex, but still incorporates abundant primary osteons.

**Radius**—The radius (Fig. S4B-D) generally resembles the humerus in its overall cross-sectional profile, but with a thicker bone wall and more diminutive marrow cavity (*RBT*, 35-40%; *K*, 0.15-0.26) as is typical of the epipodial elements. The small central cavity is lined by a thick, multi-lamellar layer of endosteal bone. The total diameter of the cavity varies between 0.25-0.50 mm. The cortical bone is composed of a predominantly parallel-fibered bone matrix with sparse primary osteons. The lacunocanalicular network was also somewhat sparse and osteocytes became increasingly lenticular in shape toward the outer margin of the bone. The primary osteons are mainly oriented longitudinally, but with a few reticular connections. They are concentrated in the inner half to two-thirds of the cortex and become very sparse in the outer cortex, where the vasculature mainly consists of a few simple canals. The overall degree of vascularization is quite low (%*CV*, 3.4-4.8; mean *POD*, 46-47 µm). Growth marks are better recorded in the epipodials than in the humerus of BP/1/4404. At least three (and possibly four) growth marks are preserved throughout the cortex, although their precise number is difficult to count due to their association with fine incremental lines. Association of incremental lamellar bone and a lack of sharply defined cement lines indicate that these waves of growth marks represent annuli (as opposed to LAGs) marking periodic decelerations in growth rather than cessations. The growth marks become more closely spaced in the subperiosteal region, but do not form an avascular EFS (as suggested by the presence of simple canals).

**Ulna**—Apart from its more flattened cross-sectional shape, the ulna (Fig. S4E, F) is near identical to the radius in its histological profile and tissue structures. The cortical bone wall is similarly thick (*RBT*, 39%; *K*, 0.20-0.22) and dominated by parallel-fibered bone with longitudinal primary osteons and cyclic growth marks (annuli). Osteocyte lacunae are sparse and strongly lenticular in shape in the outer cortex as in the radius. The cortex is also only modestly vascularized (%*CV*, 3.0-4.7; mean *POD*, 39-43 µm). Some osteons in regions where the bone wall is relatively thinner have the gross appearance of secondary osteons (Fig. S4E). However, this is mainly due to their large size and multi-lamellar infilling. Close inspection reveals that there is no cement line surrounding these osteons, and that the surrounding fabric of the interosteonal ECM also conforms to their shape. Secondary osteons are therefore absent.

*Mirotenthes digitipes*

(Fig. S5)

**General**—SAM-PK-K6511 (humerus, radius, ulna, femur, tibia, fibula) from Steenkampshoek (Upper Permian, *Cistecephalus* AZ). As in *Hofmeyria* (BP/1/4404), limb bone shafts are thick-walled for their small size, and the cortex is composed of moderately vascularized FLB with primary osteons (and simple canals) and a predominantly parallel-fibered interstitial ECM. Cyclic growth marks are present throughout the cortex, especially toward the outer periphery. The subperiosteal region of some elements preserves more parallel-fibered or lamellar bone with sparse vascular canals. Secondary osteons are absent in all elements.

**Humerus**—The humerus shaft (Fig. S5A) exhibits a compact cross-sectional profile with a thick bone wall (*RBT*, 32; *K*, 0.31) and a small central cavity with a slightly irregular endosteal margin and some trabecular structures occupying the cavity. The cortex incorporates numerous primary osteons within a predominantly parallel-fibered interstitial ECM. Osteocyte lacunae were well-concentrated and became increasingly lenticular toward the periphery of the bone. The vasculature is very simple in its organization, mainly consisting of many sparsely distributed longitudinal primary osteons and occasional simple canals (%*CV*, 5.4; mean *POD*, 54 µm). The primary osteons occasionally (though infrequently) bear radial and circular anastomoses, especially deeper in the cortex. Closer to the periphery, primary osteons become fewer and give way to simple canals of varying orientations. Growth marks are more clearly delineated in this humerus than in that of BP/1/4404, and are best visualized in normal (non-polarized) light. Three evenly spaced growth marks are present in the mid-cortex, and a fourth in the subperiosteal region. As in BP/1/4404, growth marks are preserved in the form of annuli, and are associated with faint incremental lines and waves of birefringent parallel-fibered bone when viewed under polarized light. There is no EFS.

**Radius**—The radius (Fig. S5B) has a similarly thickened bone wall at midshaft (*RBT*, 34; *K*, 0.33) with a small but relatively open central cavity. The cavity is unobstructed by trabecular or cancellous structures, instead lined by a smooth layer of endosteal lamellar bone, and is approximately 0.80 mm in diameter. The cortex is formed primarily by parallel-fibered bone that is modestly vascularized by sparse primary osteons and simple canals (%*CV*, 3.5; mean *POD*, 38 µm). The lacunocanalicular network is poorly preserved in this element, but osteocyte lacunae apparently became more sparse in the outer cortex. Most of the vascular network is localized within the inner half of the cortex (but with a few simple canals present in the outer half). At least five annuli are present, more distinct than in the humerus, and are very closely spaced in the outer cortex forming an EFS-like structure that is nearly avascular, but not acellular.

**Ulna**—The histological profile of the ulna (Fig. S5C) is near identical to that of the radius, except for its more flattened, ellipsoidal shape in section. The bone wall is thick (*RBT*, 32; *K*, 0.42), the cortex is composed of parallel-fibered bone with many small primary osteons (%*CV*, 2.0; mean *POD*, 41 µm), and at least four or five growth marks (annuli) are present throughout the cortex. Osteocyte lacunae became increasingly sparsely distributed in the outer cortex.

**Femur**—The femur midshaft (Fig. S5D) resembles the humerus in its overall cross-sectional profile and distribution of histological features, although it is slightly thinner-walled than any of the forelimb elements (*RBT*, 25; *K*, 0.42). There is a wide, open marrow cavity with some trabeculae and erosion cavities forming within the surrounding deep cortex. A thin layer of lamellar bone lines the endosteal margin of the cortex. The deep cortex is formed by moderately vascularized FLB, consisting of a parallel- and woven-fibered matrix with longitudinally oriented primary osteons. The lacunocanalicular network is sparse and osteocytes became more lenticular in shape in the outmost cortex. The primary osteons are more consistently longitudinal than in the humerus, with fewer anastomoses, but the overall degree of vascularization is comparable (%*CV*, 3.6; mean *POD*, 58 µm). Primary osteons become fewer in the outer cortex, but are still present with few simple canals. Growth marks are present throughout the cortex, but are best seen in the outer regions where primary osteons are less densely concentrated and parallel-fibered bone dominates. There are at least four growth marks, which become progressively closer spaced toward the periphery as in the humerus. There is no EFS in this element.

**Tibia**—The tibia (Fig. S5E) exhibits an irregular, slightly figure-8 cross-sectional shape and a relatively thick bone wall (*RBT*, 31; *K*, 0.31) as in the other epipodial elements. The marrow cavity, however, is also irregular in shape with coarse trabeculae and occasional small resorption cavities forming along the endosteal margins. The cortex is formed by parallel-fibered bone with small, longitudinally and radially oriented primary osteons that are most concentrated in the deep cortex. The lacunocanalicular network is too poorly preserved for adequate description. The overall degree of vascularization is fairly modest (%*CV*, 3.1; mean *POD*, 38 µm). Numerous growth marks are preserved in the outer cortex, especially near the subperiosteal region where they form a nearly avascular EFS-like structure as in the radius.

**Fibula**—The fibula (Fig. S5F), apart from being more triangular in cross-section, resembles the tibia in its overall histological profile. It has a thick bone wall at midshaft (*RBT*, 32; *K*, 0.39) with some trabecular architecture in the marrow cavity. The cortex is formed by parallel-fibered bone with few longitudinally oriented primary osteons, and is poorly vascularized (%*CV*, 3.9; mean *POD*, 32 µm). Osteocyte lacunae are poorly preserved as in the tibia. Growth marks are faint, but an avascular EFS-like structure forms a distinctive collar around the bone.

Whaitsiidae

*Theriognathus microps*

(Fig. S6)

**General**—BP/1/719 (femur) from Swaelkrans, Murraysburg District (Upper Permian, *Dicynodon* AZ); NMQR 3375 (femur) from Tafelkop 712, Bloemfontein District (Upper Permian, *Dicynodon* AZ). The histological structure of the radius in *Theriognathus* has previously been described by Ricqlès (1969) (as ‘*Notosollasia’*). The specimen was recovered from the Doornplaats locality (*Dicynodon* AZ) where this taxon occurs abundantly. Based on the author’s detailed descriptions and photographic plates, the structure of the radius midshaft resembles that of the hofmeyriids described above in having an extremely thick bone wall and reduced central cavity (Ricqlès, 1969). The vascular motif was primarily longitudinal and radial. Secondary osteons are absent in all studied specimens.

**Femur**—Two femora are described here to supplement the description by Ricqlès (1969). It is worth noting that the femur of *Theriognathus* is unique in its strongly golf club-shaped head that fits into a deep acetabulum, and a bowed shaft that orients the distal condyles directly ventrally, placing the lower leg under the hip in a semi-erect stance. The midshaft is rounded in cross-section and bears a relatively thick wall (*RBT*, ~20-24%; *K*, ~0.51-0.59) compared to the hind limbs of other therocephalians, possibly related to impact loading imposed by this orientation. The thick cortical bone wall is composed of cyclically deposited FLB with numerous primary osteons in a parallel- and woven-fibered matrix. Osteocyte lacunae are generally numerous, haphazardly distributed, and globular in shape within growth zones, but more lenticular and ordered near growth marks. In the small subadult specimen sampled here (NMQR 3375), primary osteons are abundant and take on a predominantly radial orientation, but with a few longitudinal osteons arranged in radial rows or bearing radial and reticular ramifications. Numerous growth marks (LAGs) record a cyclic growth pattern, with five irregularly spaced growth marks punctuating the cortex in NMQR 3375, followed by a thick, well vascularized growth zone in the outermost cortex indicating a brief ‘spurt’ prior to death. In the larger femur (BP/1/719) there is a lesser degree of radial vasculature, the primary osteons being almost exclusively longitudinal (although with some reticular anastomoses in the outer cortex). In general, the cortex bears a moderate degree of vascularization (%*CV*, 6.2-6.8; mean *POD*, 65-87 µm). BP/1/719 preserves at least four growth marks, although its early growth record has been lost by formation of resorption cavities in the perimedullary region. The number and spacing of growth marks suggests some degree of plasticity in the growth style of *Theriognathus*.

BAURIOIDEA

Ictidosuchidae

*Ictidosuchoides longiceps*

(Fig. S7)

**General**—BP/1/75 (humerus), from Sondasgriviershoek, Cambedoo, Graaff-Reinet District (Upper Permian, *Cistecephalus* AZ); BP/1/4092 (humerus, radius, ulna) from Matjiesfontein, Victoria West (Upper Permian, *Cistecephalus* AZ); SAM-PK-K8659 (humerus, radius, femur, tibia, fibula) from Wilgerboskloof, Prince Albert District (Upper Permian, *Tropidostoma* AZ); SAM-PK-K10423 (femur, tibia, fibula) from Doornplaats, Graaff-Reinet District (Upper Permian, *Dicynodon* AZ). The specimens present a variety of sizes and ages, ranging from the small skeleton SAM-PK-K8659 (BSL = 101 mm), which bears neurocentral sutures, to the largest known specimen BP/1/4092 (BSL = 200 mm). Mid-diaphyseal sections reveal somewhat thinner bone walls in *Ictidosuchoides* (and perhaps baurioids in general) than in other therocephalians with *RBT*s generally ranging from 14-26% (rather than 25-40% in akidnognathids and hofmeyriids). The cortices are formed by moderately vascularized parallel-fibered bone with reticular and longitudinal primary osteons, and punctuated by cyclic growth marks. There are no secondary osteons.

**Humerus**—The humerus (Fig. S7A, G, H) is a slender bone with a well-defined shaft having a subcircular cross-sectional profile. The overall compactness of the bone is low with a fairly thin bone wall (*RBT*, ~19-22%; *K*, ~0.56-0.64). The marrow cavity is large and relatively hollow with little cancellous structure (a few trabeculae are present in proximal shaft of BP/1/75). The endosteal perimedullary margin is smooth at the midshaft. Resorption cavities are near absent in the perimedullary region. The remaining cortex generally consists of reticular FLB in a parallel- and woven-fibered matrix. Osteocyte lacunae are generally numerous and disorganized with a globular shape in growth zones, but more lenticular and ordered near growth marks as in the femur of *Theriognathus*. The reticular primary osteons occasionally bear circular anastomoses that give a subplexiform appearance in localized regions of the cortex. The osteons are quite small, but the bone is moderately well vascularized overall (%*CV*, ~7.3-11.1; mean *POD*, ~45-60 µm) with most of the osteons being concentrated within the deep cortex. Cyclic growth marks are faint but present, usually in the form of annuli with associated waves of ‘streaky’ parallel-fibered bone as in the hofmeyriids. The number of growth marks varies, with a single subperiosteal annulus in the small SAM-PK-K8659, two faint annuli preserved in the medium BP/1/75, and three annuli in the largest BP/1/4092. Growth apparently did not fully cease in the largest individual, but more parallel-fibered and lamellar bone formation occurred in the outer half of the cortex, with more lamellar fibers and flattened osteocyte lacunae appearing after the third (outermost) annulus.

**Radius**—The radius (Fig. S7B, I), like the humerus, bears a slender shaft having a bone wall of moderate thickness (*RBT*, ~21-24%; *K*, ~0.52-0.58), contrasting with the forelimb epipodials of other non-baurioid therocephalians. The endosteal perimedullary margin is relatively smooth at the midshaft and there are few trabeculae within the marrow cavity. The cortical bone wall is composed of FLB with small, longitudinal primary osteons and the interstitial ECM apparently became increasingly parallel-fibered toward the outer periphery. Osteocyte lacunae are generally numerous and disorganized, and globular in shape, but became sparser and more lenticular near growth marks and in the outer cortex of large individuals. Osteons are very small and mostly longitudinal, but exhibit occasional reticular motifs. The transition to parallel-fibered bone occurred early during the bone’s growth, as it is evident in the smallest specimen (SAM-PK-K8659) where bundles of parallel-fibered bone were deposited just prior to formation of a subperiosteal annulus (as in the humerus). The larger BP/1/4092, retains a similar pattern, with alternating waves of deposition of parallel-fibered bone concentrated near three faint annuli moving toward the outer periphery. The size and concentration of primary osteons also decreases toward the periphery and overall vascularity is moderate to low (%*CV*, ~4.2-11.1; mean *POD*, ~38-62 µm). No more than three growth marks are observed throughout the cortex in the largest specimens and there is no EFS.

**Ulna**—The ulna (BP/1/4092) forms a flattened diaphysis with an oval cross-section and a bone wall that is of moderate thickness (*RBT*, 26%; *K*, 0.54). The marrow cavity is large with an irregular endosteal margin and several trabeculae present within the cavity. The structure of the cortex closely resembles the radius, being composed of FLB with small, longitudinal and reticular primary osteons and occasional streaks of parallel-fibered bone when viewed under polarized light. As in the radius, osteocyte lacunae are disorganized and globular, but became sparser and more lenticular near growth marks and in the outer cortex. Frequent reticular canals are present throughout the preaxial region of the cortex, forming primary osteons of moderate size in a woven- and parallel-fibered matrix incorporating dense clusters of globular osteocyte lacunae. The overall %*CV* is 8.3 and the mean *POD* is 52 µm. Growth marks, though present, are difficult to distinguish due to poor preservation, and a complete count cannot be performed. At least one distinct annulus can be observed in the outer two-thirds of the cortex, but there were likely more given those recorded in the radius and humerus of the same individual.

**Femur**—The femur (Fig. S7D, E) bears a long, straight shaft that is thinner walled than in any of the forelimb elements (*RBT*, 14%; *K*, 0.71). The marrow cavity is very large with an irregular boundary of coarse, trabecular bone between it and the more compact cortex. In the small SAM-PK-K8659, prominent trabecular structures are present along the perimedullary margins and large cavities (~60-100 µm in diameter) not interpreted as erosion cavities are surrounded by highly woven bone before giving way to a very thin layer of woven- and parallel-fibered bone with primary osteons in the outmost region of the mid- and distal shaft. The highly woven texture preserved in the perimedullary region resembles the endochondral scaffold of perinatal bone in mammals and some archosaurs (Horner et al., 2001). In general, the more peripheral primary osteons are fairly small and have both longitudinal and radial orientations (%*CV*, 4.7; mean *POD*, ~38-65 µm). Parallel-fibered bone became more prominent in the outermost regions of the midshaft in SAM-PK-K10423. The lacunocanalicular network is poorly preserved in both femur specimens. A possible subperiosteal annulus was formed in SAM-PK-K10423, but no growth marks were otherwise detected in either specimen. Some obliquely oriented Sharpey’s fibers are preserved on the ventral surface of the distal shaft. No EFS was observed in the femur.

**Tibia**—The tibia (Fig. S7C) exhibits a structure similar to the radius and ulna. It bears a flattened cross-section with a modest bone wall (*RBT*, ~16-23%; *K*, ~0.49-0.63). The small SAM-PK-K8659 records the same transition between the porous, woven perimedullary bone and parallel-fibered cortical bone as in the radius and femur. The cortex is composed of parallel-fibered bone with small longitudinal and reticular primary osteons and some intervening Sharpey’s fibers preserved in the anterior region in SAM-PK-K8659. The cortex is poorly vascularized (%*CV*, 2.8-4.3; mean *POD*, 39-43 µm). Two annuli are preserved in the cortex of SAM-PK-K10423. There is no EFS.

**Fibula**—The fibula (Fig. S7F) exhibits a subcircular cross-section, and resembles the tibia in its histological profile. The bone wall is moderately thin at the midshaft (*RBT*, ~19-22%; *K*, ~0.47-0.59) and retains some of the porous, woven architecture in the perimedullary region as observed in the radius, femur, and tibia of SAM-PK-K8659. The transition to the compact cortex is marked by a sharp cement line, followed by deposition of sparsely vascularized parallel-fibered bone as in the tibia. The overall vascularization is fairly modest (%*CV*, ~3.8-4.7; mean *POD*, 40 µm). The primary osteons are longitudinally oriented and there are a few simple canals. Growth marks are not distinguishable in the fibula and there is no EFS.

Lycideopidae

*Tetracynodon darti*

(Fig. S8)

**General**—NMQR 3745 (humerus), from Bethal, Bethulie District (Lower Triassic, *Lystrosaurus* AZ); UCMP 78395 (humerus, radius, ulna, femur), from Wonderkrantz, Harrismith District (Lower Triassic, *Lystrosaurus* AZ); UCMP 78396 (humerus, femur, fibula), from Wonderkrantz, Harrismith District (Lower Triassic, *Lystrosaurus* AZ). Sigurdsen et al. (2012) briefly described bone histology in the humerus and femur of *Tetracynodon* with the purpose of determining relative age. The bone walls are generally thin (as in other baurioids), especially in the femur. In all elements, the mid-diaphyseal cortex consists of moderately vascularized woven- and parallel-fibered bone, usually with longitudinal primary osteons. The cortices are, in general, less vascularized than in its Permian counterpart *Ictidosuchoides*. Growth marks are often absent

indicating a relatively short growth period in sampled specimens, although there may be one or two preserved in the mid- to outer cortex in some specimens. Secondary reconstruction was limited and there are no secondary osteons.

**Humerus**—The humerus (Fig. S8A-C) exhibits an intermediate bone wall thickness (*RBT*, ~21-24%; *K*, ~0.49-0.56) and preserves well the general pattern of growth. The large, open marrow cavity is bordered by smooth endosteal lamellar bone, demarcating the transition to the cortex. There are occasional trabecular structures localized in the proximal shaft near the deltopectoral crest. The crest is more restricted along the shaft than in earlier (non-baurioid) therocephalians, such that the cross-section is more subcircular than tear-shaped. The cortex consists of moderately vascularized woven- and parallel-fibered bone incorporating longitudinal primary osteons with occasional reticular anastomoses (%*CV*, ~4.7-8.1; mean *POD*, ~36-42 µm). The deep cortex maintains a clear fibrolamellar structure with densely-packed primary osteons within a woven-fibered interstitial ECM. The lacunocanalicular network of the deep cortex is generally disorganized with large, globular osteocyte lacunae, but quickly transitioned to more ordered, lenticular osteocytes in the outer half of the cortex. The cortical bone of *Tetracynodon* bears the highest proportion of ordered, lenticular osteocytes than any studied therocephalian. In NMQR 3745, this pattern gave way to more parallel-fibered bone with lenticular osteocytes in the outer half of the cortex. This transition is demarcated by a single LAG, although a second faint annulus associated with bundles of parallel-fibered bone is present in the outer cortex. Other specimens (e.g., UCMP 78395, 78396) show more consistent parallel-fibered bone deposition. The outer cortex incorporates lamellar tissue with fewer vascular canals and is punctuated by a single LAG near the subperiosteal region. The LAG is succeeded by a thin band of parallel-fibered and lamellar bone (varying from ~50 to 100 µm) with few simple canals located near the periosteal margin, indicating that although growth had attenuated it had not fully ceased. No EFS is present in any of the specimens.

**Radius**—The radius (UCMP 78395) (Fig. S8D) has a subcircular cross-section at midshaft and bears a slightly thicker bone wall than in the humerus (*RBT*, 27%; *K*, 0.43). The perimedullary region bears a relatively smooth ring of endosteal lamellar bone marking the border with the marrow cavity. A few resorption cavities are present in the perimedullary region, especially near the postaxial region of the bone. This region also preserves abundant Sharpey’s fibers throughout much of the cortex. The cortex is predominately formed by poorly vascularized parallel-fibered bone with primary osteons and simple canals (%*CV*, 1.9; mean *POD*, 42 µm). Osteocyte lacunae are globular in shape and haphazardly arranged, but are sparsely distributed and became more lenticular in some areas of the outer cortex. The sparse vascular canals bear an exclusively longitudinal orientation. Growth marks appear to be absent in the studied specimen.

**Ulna**—The ulna (UCMP 78395) is similar to the radius in its histological profile, although it is more flattened and bears more trabecular architecture in the marrow cavity. The bone wall is somewhat thicker (*RBT*, 31%; *K*, 0.33) and the transition between the marrow cavity and the cortex is irregular and less abrupt. Large resorption cavities are present in the perimedullary region and the inner half of the cortex exhibits abundant woven-fibered tissue. The lacunocanalicular network is sparse as in the radius, with osteocytes becoming fewer and more lenticular in the outer cortex. The vasculature becomes extremely sparse in the outer cortex (%*CV*, 2.8; mean *POD*, 38 µm) where there is also more parallel-fibered bone. Vascular canals are almost exclusively longitudinal as in the radius, although there are a few reticular canals. Growth marks are apparently absent in the ulna and there is no EFS.

**Femur**—The femur (Fig. S8E, F) has a slender shaft that is subcircular in section, exposing a thin bone wall and a large, open marrow cavity (*RBT*, 17%; *K*, 0.66). The transition between the marrow cavity and the cortex is abrupt, demarcated by a smooth layer of endosteally deposited lamellar bone. The composition of the cortex is similar to that of the humerus, although there is more woven-fibered tissue. As in the humerus, the osteocyte lacunae are frequently lenticular in shape, but are somewhat less ordered. The cortex is dominated by FLB having woven- and parallel-fibered interstitial ECM and numerous small primary osteons (%*CV*, ~4.3-5.0; mean *POD*, ~39-42 µm). The primary osteons are both longitudinal and reticular. In UCMP 78396, there is a transition to parallel-fibered and lamellar bone in the outermost cortex that is associated with a possible annulus. This transition is also associated with a decrease in the size and concentration of vascular canals toward the subperiosteal surface. Growth marks are otherwise absent from the cortex and there is no true EFS. In addition to cross-sectional sampling, longitudinal sections were possible across the head of the femur in UCMP 78396, allowing brief discussion of longitudinal growth. Most of the subarticular bone formed columns of longitudinally oriented trabeculae that are preserved throughout much of the epiphyseal region. The well developed articular surface of the femoral head bears a remarkably thin (∼50 microns) layer of calcified cartilage, further supporting that longitudinal growth was nearing completion but had probably not fully ceased. There is no evidence of a secondary ossification center.

**Fibula**—The fibula (Fig. S8G) is a long, thin bone that is subcirular in section at midshaft and has a bone wall of moderate thickness (*RBT*, 25%; *K*, 0.53). The marrow cavity is lined by smooth endosteal lamellar bone and there are few resorption cavities formed in the perimedullary region. The cortex is composed of modestly vascularized FLB having a woven- and parallel-fibered interstitial ECM and numerous small primary osteons (%*CV*, 2.3; mean *POD*, 35 µm). The osteons are almost exclusively longitudinal but with very few reticular anastomoses. The bone maintains a relatively disorganized appearance all the way to the subperiosteal region, with abundant woven-fibered texture and globular osteocyte lacunae. There are no growth marks apparent in the cortex and no EFS.

Scaloposauridae

*Scaloposaurus constrictus*

(Fig. S9)

**General**—SAM-PK-K4638 (humerus), from Vegtlager 801, Wagendriftdam (Lower Triassic, *Lystrosaurus* AZ).

**Humerus**—A single humerus was available for sectioning. However, poor preservation and slight crushing have distorted aspects of the specimen’s cross-sectional shape. The shaft is short but slender, and bears a subcircular cross-section as in other baurioids. The bone wall appears to have been relatively thin, with a large marrow cavity having a few trabeculae as in other baurioids. The trabeculae were formed around the perimedullary region but tended to be localized toward the deltopectoral crest. There appears to have been some endosteal erosion and none of the internal endochondral bone (unlike that which was reported above in small *Ictidosuchoides*) is retained despite the small size of the individual. The cortex preserves the intramembranously-deposited subperiosteal primary bone. The overall tissue texture is fibrolamellar, having a woven-fibered interstitial ECM that incorporated primary osteons. The osteons are relatively small and longitudinally oriented with a few reticular canals. The overall degree of vascularization is somewhat moderate (%*CV*, 6.6; mean *POD*, 62 µm). Growth marks are absent from the inner cortex, although the texture of the interstitial ECM became more parallel-fibered to lamellar in the outermost cortex with a subperiosteal collar of lamellar bone (~30-40 µm in thickness). Although osteocyte lacunae are generally large, globular and highly concentrated through much of the cortex, they take on an abrupt lenticular appearance and more ordered arrangement within the outer collar of lamellar bone. Although the subperiosteal structure indicates deceleration of growth prior to death, it does not exhibit characteristics of an EFS. Moreover, the full extent of this zone around the periphery of the bone is uncertain due to imperfect preservation and loss of some subperiosteal bone during preparation. Secondary osteons were absent.

Bauriidae

*Microgomphodon oligocynus*

(Fig. S10)

**General**—NMQR 3189 (humerus, femur, tibia, fibula), from Eerstegeluk 131, Bethlehem (Middle Triassic, *Cynognathus* AZ). King (1996) described the morphology of the studied specimen. It consists of a small, disarticulated skeleton preserved within a burrow. In general the limb bones are relatively slender with thin bone walls, bearing large, open marrow cavities as in other baurioids. The cortex consists of FLB with longitudinal, radial, and circular (laminar) primary osteons. Absence of cyclic growth rings indicates fast, sustained growth in the individual. There are no secondary osteons in any of the studied elements.

**Humerus**—The humerus (Fig. S10A) has a long, slender shaft with a subcircular cross-section. The bone wall is fairly thin as in other baurioids (*RBT*, 21%; *K*, 0.61) and borders a wide, open marrow cavity that is unobstructed by trabecular structures. A few trabeculae are present in the perimedullary region of the proximal shaft near the deltopectoral crest. The cortex is formed by FLB with a woven- and parallel-fibered matrix and longitudinal and reticular primary osteons (with localized circular canals and abundant parallel-fibered bone along the ventral cortical wall). Osteocyte lacunae are abundant, globular in shape and haphazardly arranged throughout the entire cortex. The cortex is moderately well-vascularized (%*CV*, 8.8; mean *POD*, 43 µm). Growth marks were apparently absent and there is no EFS.

**Femur**—The histological profile of the femur midshaft (Fig. S10B, C) somewhat resembles that of the humerus, bearing a similarly thin wall (*RBT*, 18%; *K*, 0.61). The marrow cavity is wide, circular and unobstructed by trabeculae, with an abrupt transition to the cortex. There are few resorption cavities along the perimedullary margins. Unlike the humerus, the entire cortex is composed of reticular FLB. The interstitial ECM is predominantly woven-fibered, being disorganized with high concentrations of globular osteocyte lacunae as in the humerus. The reticular primary osteons are densely distributed all the way to the subperiosteal region. Overall, the cortex exhibits moderate vascularization (%*CV*, 6.1; mean *POD*, 44 µm). No growth marks are preserved and there is no EFS.

**Tibia**—The tibia (Fig. S10D) is somewhat mediolaterally compressed in section and records an irregular transition between the marrow cavity and the cortex at the midshaft. The bone wall is of moderate thickness (*RBT*, 24%; *K*, 0.51), thicker than that of the propodial elements. There are some coarse trabecular structures and a few large resorption cavities along the perimedullary margins. Like the femur, the cortex is largely formed by FLB, although the primary osteons bear a predominantly longitudinal orientation (with occasional reticular anastomoses). The globular osteocytes were densely-packed around the primary osteons, which were in turn well-concentrated throughout the cortex (%*CV*, 8.0; mean *POD*, 41 µm). No growth marks are preserved and there is no EFS.

**Fibula**—The fibula (Fig. S10E) is a long, slender bone with an oval midshaft cross-section. The bone wall is somewhat thicker than in the other studied elements (*RBT*, 29%; *K*, 0.39), and the transition to the cortex is relatively abrupt. There were no resorption cavities along the perimedullary regions. Much of the inner cortex bears large, densely packed osteocyte lacunae within a woven-fibered bone matrix. The overall tissue composition is fibrolamellar with longitudinal and occasional radial primary osteons (the latter being most obvious along the posterior region of the bone). The anterior region of the bone bears obliquely-oriented Sharpey’s fibers. The overall vascularization is fairly modest (%*CV*, 4.2; mean *POD*, 32 µm). Growth marks are not observed and there is no EFS.

**References:**

Botha-Brink J, Modesto SP. 2011. A new skeleton of the therocephalian synapsid *Olivierosuchus parringtoni* from the Lower Triassic South African Karoo Basin. *Palaeontology* 54:591–606.

Chinsamy-Turan A. 2012. *Forerunners of Mammals: Radiation, Histology, Biology.* Indiana University Press, Bloomington.

Horner J & al. 2001. Comparative osteohistology of some embryonic and perinatal archosaurs: developmental and behavioral implications for dinosaurs. *Paleobiology* 27:39–58.

Huttenlocker AK, Botha-Brink J. 2013. Body size and growth patterns in the therocephalian *Moschorhinus kitchingi* (Therapsida: Eutheriodontia) before and after the end-Permian extinction in South Africa. *Paleobiology* 39:253–277.

King GM. 1996. A description of the skeleton of a bauriid therocephalian from the Early Triassic of South Africa. *Annals of the South African Museum* 104:379–393.

Kitching JW. 1977. The distribution of the Karroo vertebrate fauna. *Bernard Price Institute for Palaeontological Research Memoir 1*. The Natal Witness Ltd.: 131 pp.

Ray S, Botha J, Chinsamy A. 2004. Bone histology and growth patterns of some nonmammalian therapsids. *Journal of Vertebrate Paleontology* 24:634–648.

Ricqlès A de. 1969. Recherches paléohistologiques sur les os longs des Tétrapodes II—Quelques observations sur la structure des os longs des Thériodontes. *Annales de Paléontologie (Vertébrés)* 55:1–52. [in French]

Stein K, Prondvai E. 2013. Rethinking the nature of fibrolamellar bone: an integrative biological revision of sauropod plexiform bone formation. *Biological Reviews of the Cambridge Philosophical Society* 2013. doi: 10.1111/brv.12041

**Supporting Figures**

**Figure S1.** Bone histology in *Lycosuchus vanderrieti* from the Middle Permian *Tapinocephalus* Assemblage Zone. **A**, SAM-PK-9084, radius midshaft, cortical fibrolamellar bone viewed at high magnification (crossed-nicols with wave plate). **B**, Same as ‘A,’ viewed under normal polarized light without wave plate. **C**, SAM-PK-9084, ulna midshaft, cortex showing growth marks and well-vascularized fibrolamellar bone viewed at low magnification (non-polarized light). Note the thick bone wall and inner coarse cancellous structure. **D**, SAM-PK-K9012, femur midshaft, dorsal cortex showing subplexiform fibrolamellar bone viewed under non-polarized light. **E**, SAM-PK-K9012, femur midshaft, posterior region of cortex showing three bands of parallel-fibered bone (blue bands denoted by arrows) representing possible growth marks, viewed at low magnification (crossed-nicols with wave plate). Arrows denote growth marks. Abbreviations: po, primary osteon.

**Figure S2.** Bone histology in Scylacosauridae from the Middle Permian *Tapinocephalus* Assemblage Zone. **A**, *Glanosuchus* *macrops*, BP/1/6228, ulna midshaft, cortical fibrolamellar bone viewed at low magnification (crossed-nicols with wave plate). **B**, Scylacosauridae indet., SAM-PK-5018, fibula midshaft close-up of secondary osteon in deep cortex (crossed-nicols with wave plate). **C**, Scylacosauridae indet., CGS R300, humerus midshaft cortex viewed at low magnification showing growth marks (crossed-nicols with wave plate). **D**, Scylacosauridae indet., BP/1/5576, ulna midshaft cortex viewed at low magnification showing growth marks (crossed-nicols with wave plate). **E**, Scylacosauridae indet., BP/1/5587, ulna midshaft cortex viewed at low magnification showing growth marks (crossed-nicols with wave plate). Arrows denote growth marks. Abbreviations: cl, cement line; Hc, Haversian canal; po, primary osteon.

**Figure S3.** Bone histology in the akidnognathid *Olivierosuchus parringtoni* from the Triassic *Lystrosaurus* Assemblage Zone. **A**, NMQR 3605, cross-sectional profile of humerus midshaft viewed at low magnification (crossed-nicols with wave plate). Note the occluded medullary region and relatively thick cortical bone wall. **B**, NMQR 3605, humerus midshaft, cortical fibrolamellar bone showing large primary osteons preceding a thin zone of parallel-fibered bone near a LAG (crossed-nicols with wave plate). **C**, NMQR 3605, humerus midshaft, cortex showing thick zone of reticular fibrolamellar bone followed by parallel-fibered bone and a LAG (crossed-nicols with wave plate). **D**, Triassic *Moschorhinus* (SAM-PK-K118) humerus midshaft shown at same scale as ‘C’ for comparison (crossed-nicols with wave plate). Note the densely packed reticular and radial primary osteons and globular osteocyte lacunae. **E**, SAM-PK-K10617, femur midshaft, cortical fibrolamellar bone viewed at low magnification (crossed-nicols with wave plate). **F**, SAM-PK-K10617, femur midshaft, close-up of primary osteons and interstitial bone matrix (normal polarized light at maximum extinction). Arrows denote growth marks. Abbreviations: pfb, parallel-fibered bone; po, primary osteon.

**Figure S4.** Bone histology in the hofmeyriid *Hofmeyria atavus*. **A**, BP/1/4404, cortex of humerus midshaft viewed at low magnification (crossed-nicols with wave plate). **B**, BP/1/4404, cross-sectional profile of radius midshaft viewed at low magnification (crossed-nicols with wave plate). **C**, Same as ‘B,’ close-up of cortex showing growth marks and lamellar bone in outer cortex (crossed-nicols with wave plate). **D**, Same as ‘B,’ close-up of cortex showing longitudinal primary osteons and outer lamellar bone (crossed-nicols with wave plate). **E**, BP/1/4404, ulna midshaft cross-section viewed at low magnification (crossed-nicols with wave plate). **F**, BP/1/4404, ulna midshaft cortex viewed at high magnification, showing sharp transition to lamellar bone in outer cortex (crossed-nicols with wave plate). Brackets denote outer zone of lamellar bone with simple canals, indicating marked decrease in bone apposition. Abbreviations: pfb, parallel-fibered bone; po, primary osteon.

**Figure S5.** Bone histology in the hofmeyriid *Mirotenthes digitipes*. **A**, SAM-PK-K6511, humerus midshaft cross-section viewed at low magnification (crossed-nicols with wave plate). **B**, SAM-PK-K6511, radius midshaft cortex (crossed-nicols with wave plate). **C**, SAM-PK-K6511, ulna midshaft cortex (crossed-nicols with wave plate). **D**, SAM-PK-K6511, femur midshaft cortex showing extensive parallel-fibered and lamellar bone (normal polarized light at maximum extinction). **E**, SAM-PK-K6511, tibia midshaft cross-section viewed at low magnification (non-polarized light). **F**, SAM-PK-K6511, fibula midshaft cross-section viewed at low magnification (non-polarized light). Bracket indicates avascular outer zone. Abbreviations: pfb, parallel-fibered bone; po, primary osteon.

**Figure S6.** Bone histology in the whaitsiid *Theriognathus microps*. **A**, NMQR 3375, femur midshaft cross-section viewed at low magnification (non-polarized light). **B**, NMQR 3375, femur midshaft cortical bone viewed at medium magnification showing growth marks (arrows) (non-polarized light). **C**, same as ‘B,’ viewed at high magnification with wave plate, showing close-up of outer zone of well-vascularized fibrolamellar bone (bracket). **D**, BP/1/719, femur midshaft cortex (crossed-nicols with wave plate). Arrows denote growth marks. Abbreviations: flb, fibrolamellar bone; rc, radial canals.

**Figure S7.** Bone histology in the baurioid *Ictidosuchoides longiceps*. **A**, SAM-PK-K8659, humerus midshaft cross-section viewed at low magnification (normal polarized light). **B**, SAM-PK-K8659, radius midshaft cortex and inner cancellous bony scaffold (crossed-nicols with wave plate). **C**, SAM-PK-K8659, tibia midshaft cortex and inner cancellous bony scaffold (crossed-nicols with wave plate). **D**, SAM-PK-K8659, femur midshaft showing inner cancellous bone and outer bone compacta with a woven-fibered matrix (crossed-nicols with wave plate). **E**, SAM-PK-K10423, femur distal shaft cortex (crossed-nicols with wave plate). **F**, SAM-PK-K10423, fibula proximal shaft cortex (crossed-nicols with wave plate). **G**, BP/1/75, humerus midshaft cortex (crossed-nicols with wave plate). **H**, BP/1/4092, midshaft cross-section of large humerus viewed at low magnification (crossed-nicols with wave plate). **I**, BP/1/4092, radius close-up showing growth mark (annulus) in outer cortex (non-polarized light). Arrows denote growth marks. Abbreviations: nc, nutrient canal; pfb, parallel-fibered bone; spc, subplexiform canals; wb, woven-fibered bone.

**Figure S8.** Bone histology in the baurioid *Tetracynodon darti*. **A**, NMQR 3745, humerus midshaft cross-sectional profile viewed at low magnification (non-polarized light). **B**, UCMP 78396, humerus midshaft cortex (normal polarized light at maximum extinction). Bracket denotes outer zone of parallel-fibered and lamellar bone. **C**, UCMP 78396, humerus midshaft cortex showing outer line of arrested growth (demarcated by cement line at arrow) (non-polarized light). **D**, UCMP 78395, radius midshaft cortex and perimedullary region viewed at low magnification (crossed-nicols with wave plate). **E**, UCMP 78396, femur midshaft cortex and perimedullary region showing woven- and parallel-fibered bone (crossed-nicols with wave plate). **F**, UCMP 78396, femur midshaft cortex close-up (normal polarized light at maximum extinction). **G**, UCMP 78396, fibula midshaft cortical bone packed with longitudinal primary osteons (non-polarized light). Arrows denote growth marks. Abbreviations: elb, endosteal lamellar bone; pfb, parallel-fibered bone; po, primary osteon; Sf, Sharpey’s fibers; wb, woven-fibered bone.

**Figure S9.** Bone histology in the baurioid *Scaloposaurus constrictus*. **A**, Humerus midshaft cross-section showing fibrolamellar bone deposition followed by a thin collar of lamellar bone in the subperiosteal region (normal polarized light at maximum extinction). **B**, Humerus midshaft cortex close-up showing longitudinal primary osteons and a large nutrient canal within a woven-fibered bone matrix (crossed-nicols with wave plate). Abbreviations: lb, lamellar bone; nc, nutrient canal; po, primary osteon.

**Figure S10.** Bone histology in the baurioid *Microgomphodon oligocynus*. **A**, NMQR 3605, humerus midshaft cross-section viewed at low magnification (crossed-nicols with wave plate). Note local histovariation in the arrangements of vascular canals. **B**, NMQR 3605, femur midshaft cross-sectional profile viewed at low magnification (crossed-nicols with wave plate). **C**, Close-up of ‘B’ showing abundant reticular canals in cortex and lack of growth marks. **D**, NMQR 3605, tibia midshaft cross-sectional profile viewed at low magnification (crossed-nicols with wave plate). **E**, NMQR 3605, fibula midshaft cortex (crossed-nicols with wave plate). Abbreviations: cc, circular canal; rc, reticular canal; po, primary osteon; Sf, Sharpey’s fibers.
