# Supplementary figures and images for: Bone microstructure and the evolution of growth patterns in Permo-Triassic therocephalians (Amniota, Therapsida) of South Africa
Source: PeerJ. 2014 Apr 8;2:e325. doi: 10.7717/peerj.325 (PMC3994631; doi:10.7717/peerj.325)

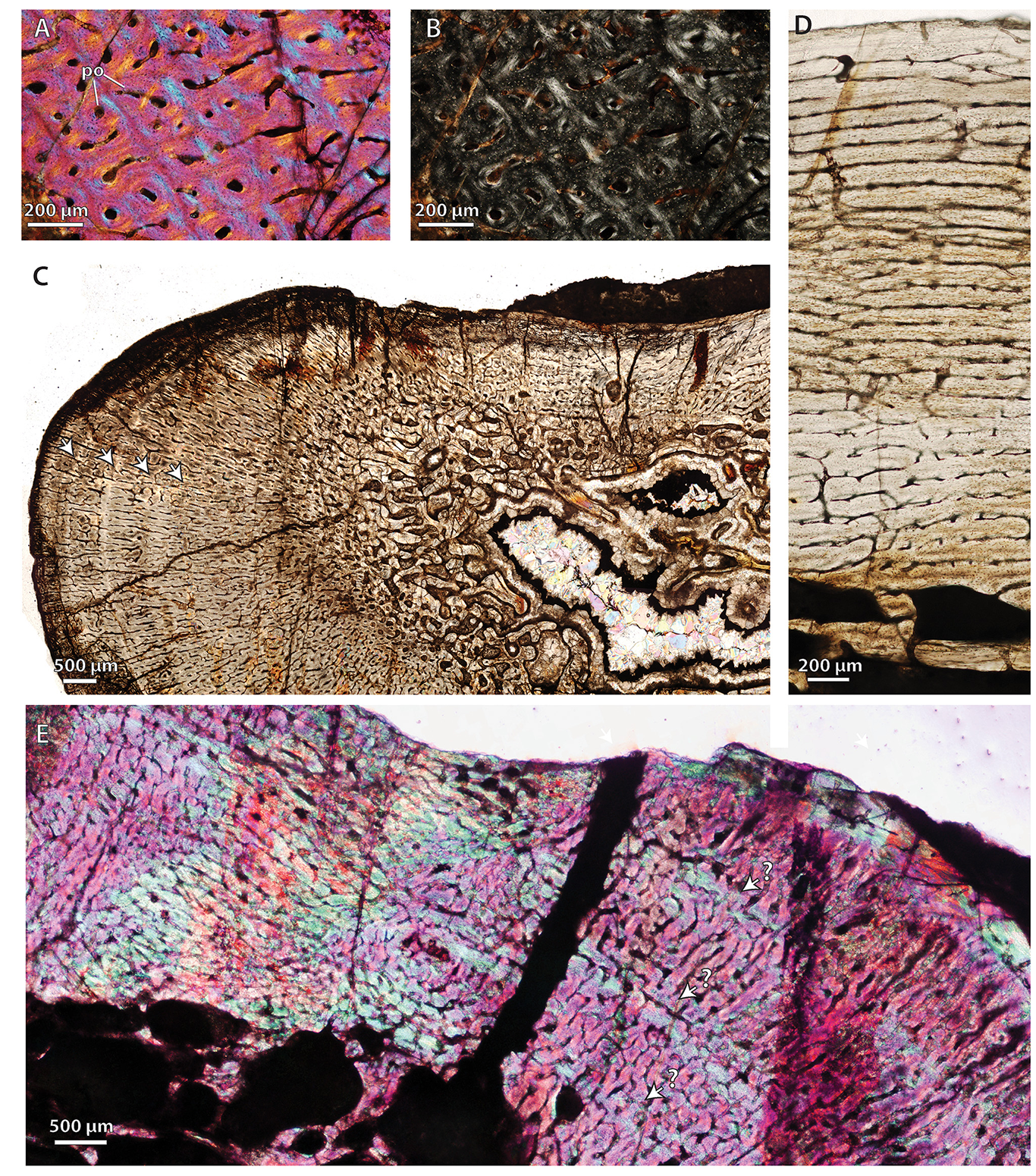

Supplement: Supplemental Information 2 — (A) SAM-PK-9084, radius midshaft, cortical fibrolamellar bone viewed at high magnification (crossed-nicols with wave plate). (B) Same as (A) viewed under normal polarized light without wave plate. (C) SAM-PK-9084, ulna midshaft, cortex showing growth marks and well-vascularized fibrolamellar bone viewed at low magnification (non-polarized light). Note the thick bone wall and inner coarse cancellous structure. (D) SAM-PK-K9012, femur midshaft, dorsal cortex showing subplexiform fibrolamellar bone viewed under non-polarized light. (E) SAM-PK-K9012, femur midshaft, posterior region of cortex showing three bands of parallel-fibered bone (blue bands denoted by arrows) representing possible growth marks, viewed at low magnification (crossed-nicols with wave plate). Arrows denote growth marks. Abbreviations: po, primary osteon. [file peerj-02-325-s002.png]

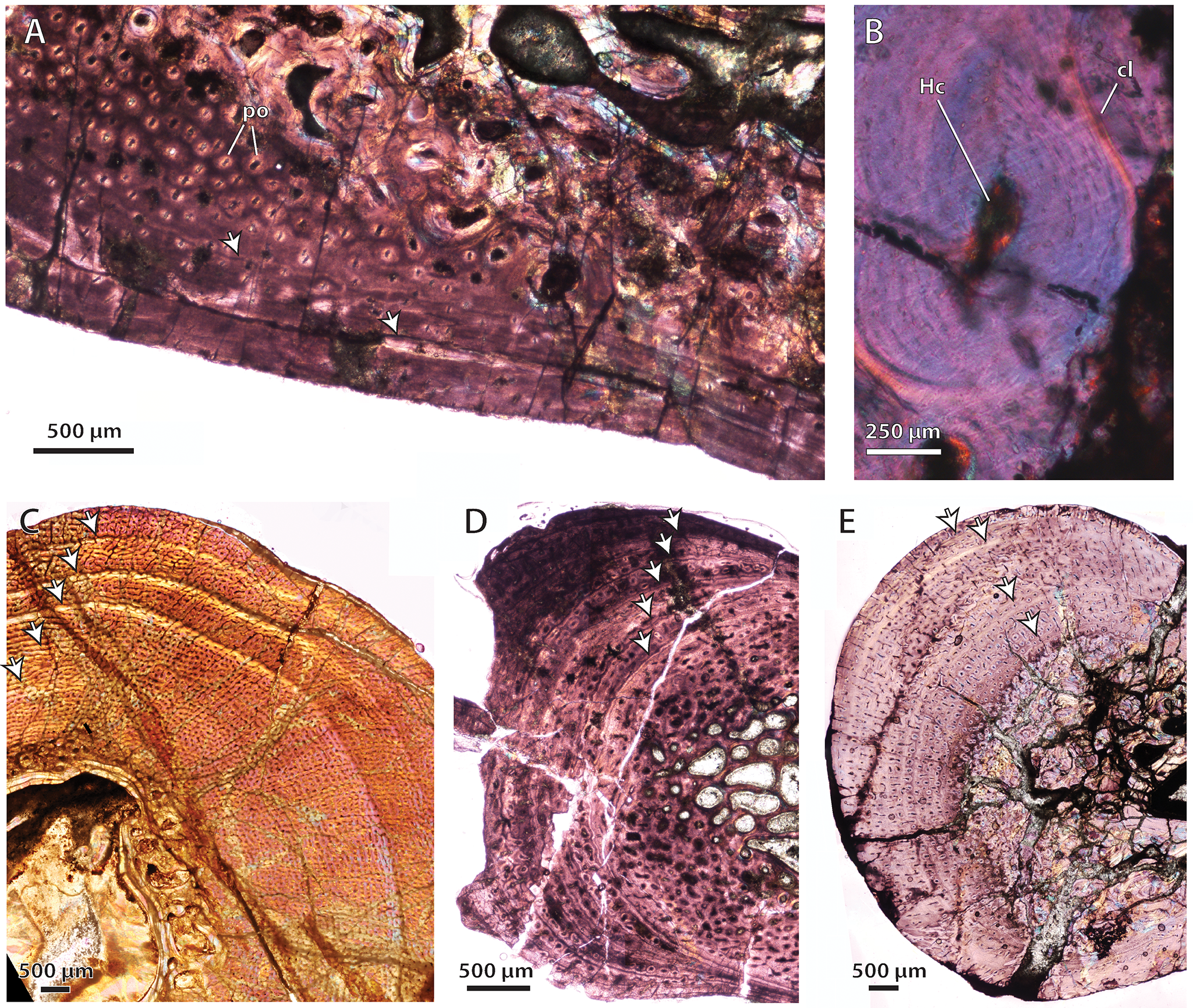

Supplement: Supplemental Information 3 — (A) Glanosuchus macrops, BP/1/6228, ulna midshaft, cortical fibrolamellar bone viewed at low magnification (crossed-nicols with wave plate). (B) Scylacosauridae indet., SAM-PK-5018, fibula midshaft close-up of secondary osteon in deep cortex (crossed-nicols with wave plate). (C) Scylacosauridae indet., CGS R300, humerus midshaft cortex viewed at low magnification showing growth marks (crossed-nicols with wave plate). (D) Scylacosauridae indet., BP/1/5576, ulna midshaft cortex viewed at low magnification showing growth marks (crossed-nicols with wave plate). (E) Scylacosauridae indet., BP/1/5587, ulna midshaft cortex viewed at low magnification showing growth marks (crossed-nicols with wave plate). Arrows denote growth marks. Abbreviations: cl, cement line; Hc, Haversian canal; po, primary osteon. [file peerj-02-325-s003.png]

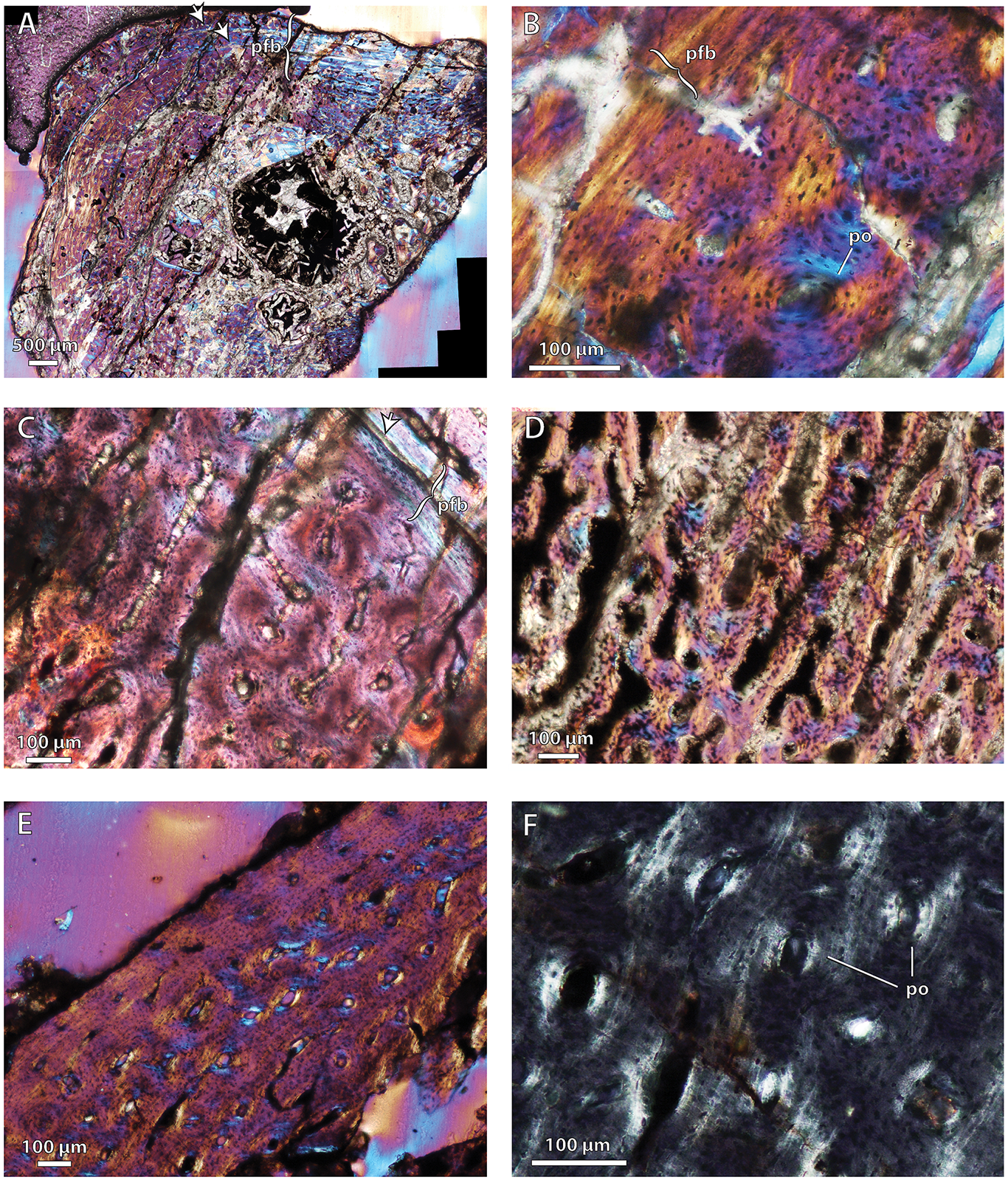

Supplement: Supplemental Information 4 — (A) NMQR 3605, cross-sectional profile of humerus midshaft viewed at low magnification (crossed-nicols with wave plate). Note the occluded medullary region and relatively thick cortical bone wall. (B) NMQR 3605, humerus midshaft, cortical fibrolamellar bone showing large primary osteons preceding a thin zone of parallel-fibered bone near a LAG (crossed-nicols with wave plate). (C) NMQR 3605, humerus midshaft, cortex showing thick zone of reticular fibrolamellar bone followed by parallel-fibered bone and a LAG (crossed-nicols with wave plate). (D) Triassic Moschorhinus (SAM-PK-K118) humerus midshaft shown at same scale as (C) for comparison (crossed-nicols with wave plate). Note the densely packed reticular and radial primary osteons and globular osteocyte lacunae. (E) SAM-PK-K10617, femur midshaft, cortical fibrolamellar bone viewed at low magnification (crossed-nicols with wave plate). (F) SAM-PK-K10617, femur midshaft, close-up of primary osteons and interstitial bone matrix (normal polarized light at maximum extinction). Arrows denote growth marks. Abbreviations: pfb, parallel-fibered bone; po, primary osteon. [file peerj-02-325-s004.png]

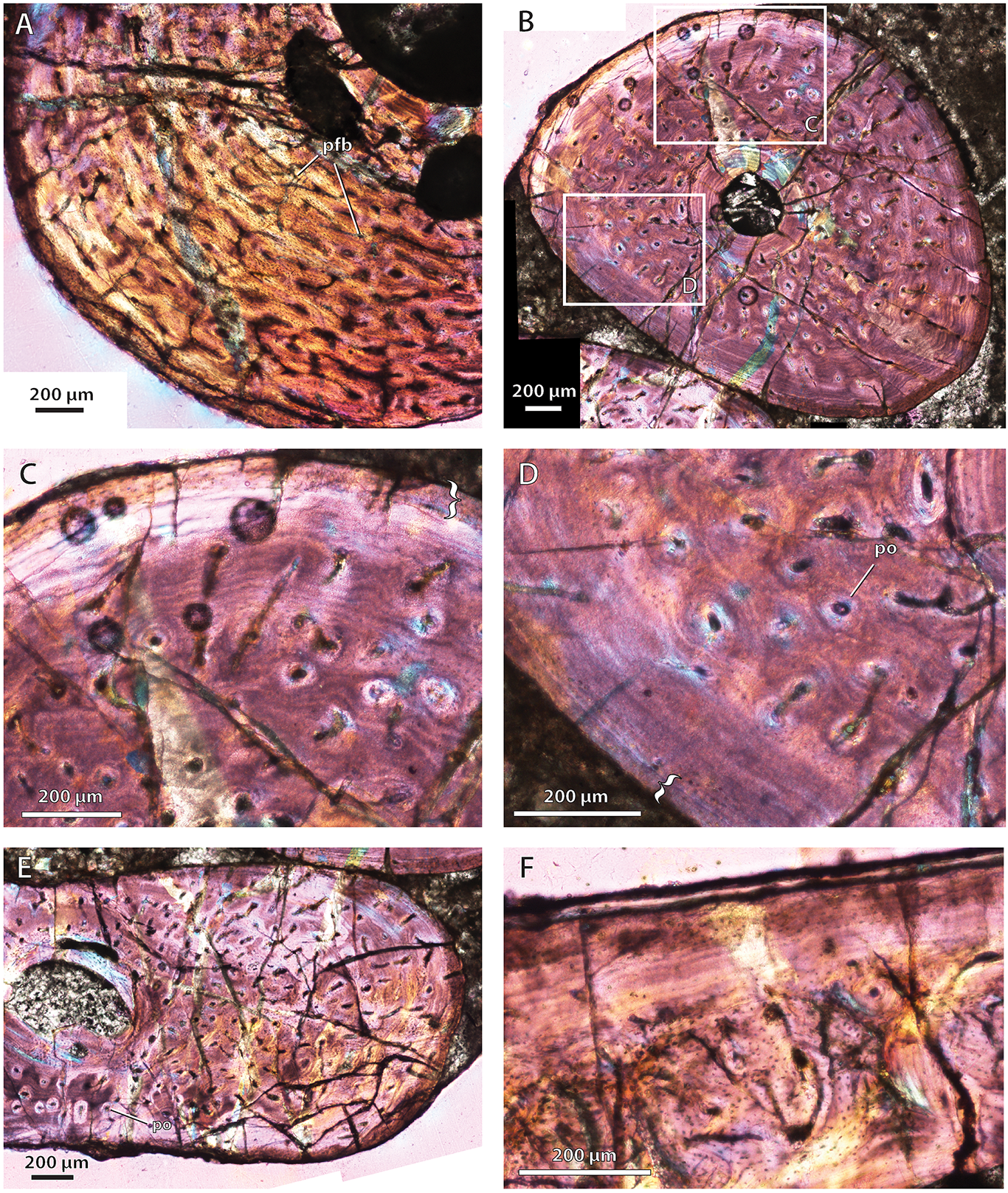

Supplement: Supplemental Information 5 — (A) BP/1/4404, cortex of humerus midshaft viewed at low magnification (crossed-nicols with wave plate). (B) BP/1/4404, cross-sectional profile of radius midshaft viewed at low magnification (crossed-nicols with wave plate). (C) Same as (B) close-up of cortex showing growth marks and lamellar bone in outer cortex (crossed-nicols with wave plate). (D) Same as (B) close-up of cortex showing longitudinal primary osteons and outer lamellar bone (crossed-nicols with wave plate). (E) BP/1/4404, ulna midshaft cross-section viewed at low magnification (crossed-nicols with wave plate). (F) BP/1/4404, ulna midshaft cortex viewed at high magnification, showing sharp transition to lamellar bone in outer cortex (crossed-nicols with wave plate). Brackets denote outer zone of lamellar bone with simple canals, indicating marked decrease in bone apposition. Abbreviations: pfb, parallel-fibered bone; po, primary osteon. [file peerj-02-325-s005.png]

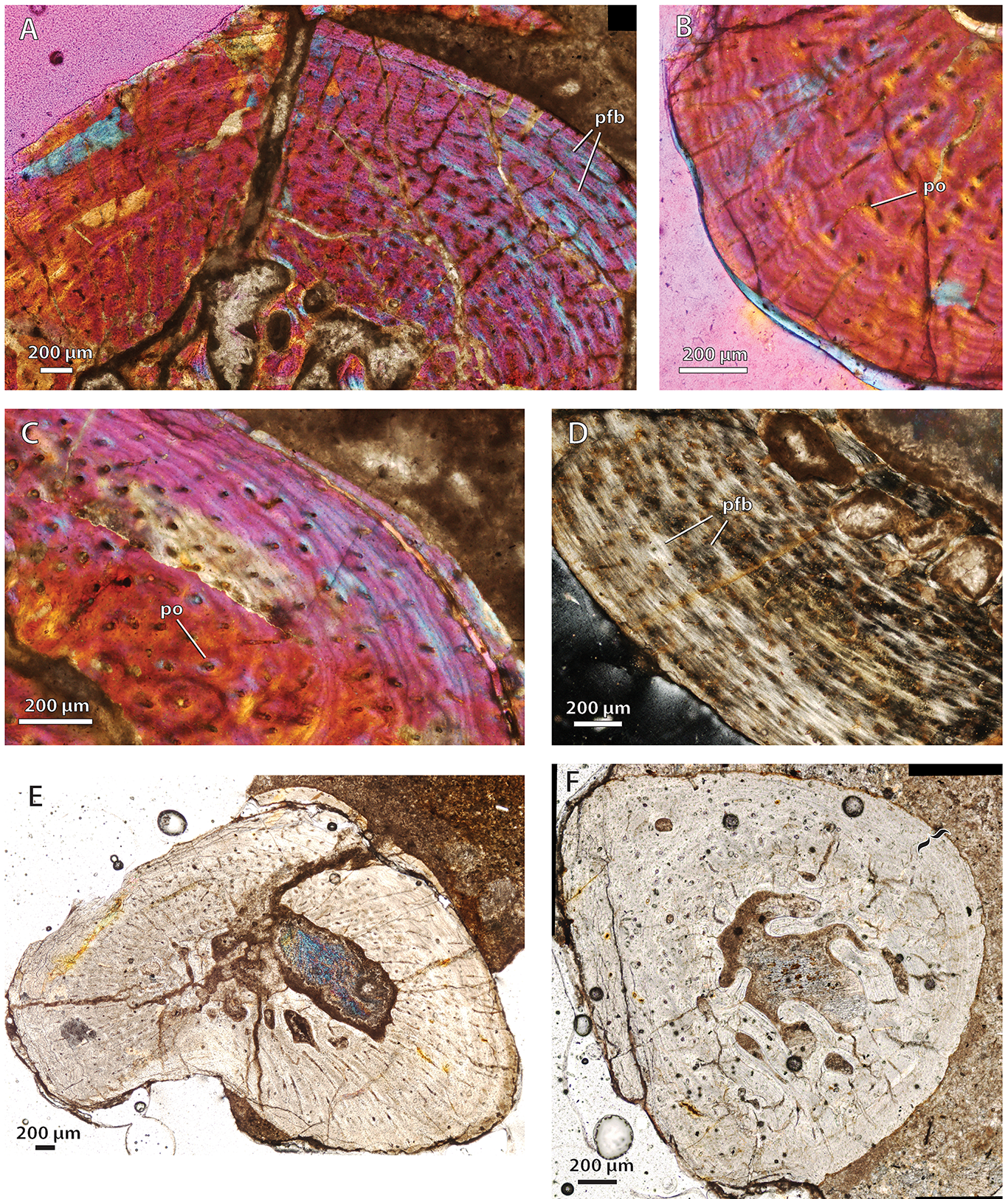

Supplement: Supplemental Information 6 — (A) SAM-PK-K6511, humerus midshaft cross-section viewed at low magnification (crossed-nicols with wave plate). (B) SAM-PK-K6511, radius midshaft cortex (crossed-nicols with wave plate). (C) SAM-PK-K6511, ulna midshaft cortex (crossed-nicols with wave plate). (D) SAM-PK-K6511, femur midshaft cortex showing extensive parallel-fibered and lamellar bone (normal polarized light at maximum extinction). (E) SAM-PK-K6511, tibia midshaft cross-section viewed at low magnification (non-polarized light). (F) SAM-PK-K6511, fibula midshaft cross-section viewed at low magnification (non-polarized light). Bracket indicates avascular outer zone. Abbreviations: pfb, parallel-fibered bone; po, primary osteon. [file peerj-02-325-s006.png]

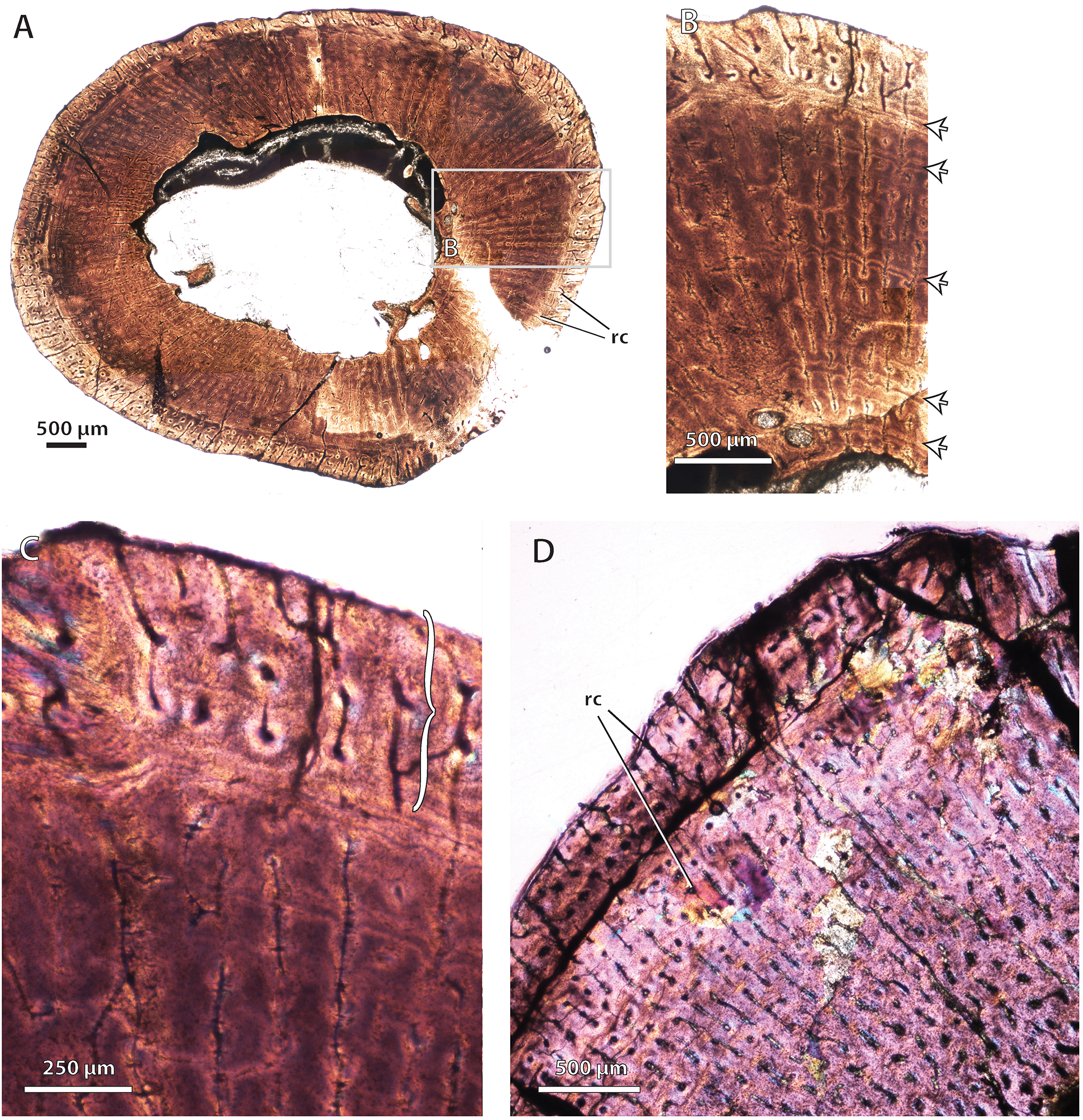

Supplement: Supplemental Information 7 — (A) NMQR 3375, femur midshaft cross-section viewed at low magnification (non-polarized light). (B) NMQR 3375, femur midshaft cortical bone viewed at medium magnification showing growth marks (arrows) (non-polarized light). (C) same as ‘B,’ viewed at high magnification with wave plate, showing close-up of outer zone of well-vascularized fibrolamellar bone (bracket). (D) BP/1/719, femur midshaft cortex (crossed-nicols with wave plate). Arrows denote growth marks. Abbreviations: flb, fibrolamellar bone; rc, radial canals. [file peerj-02-325-s007.png]

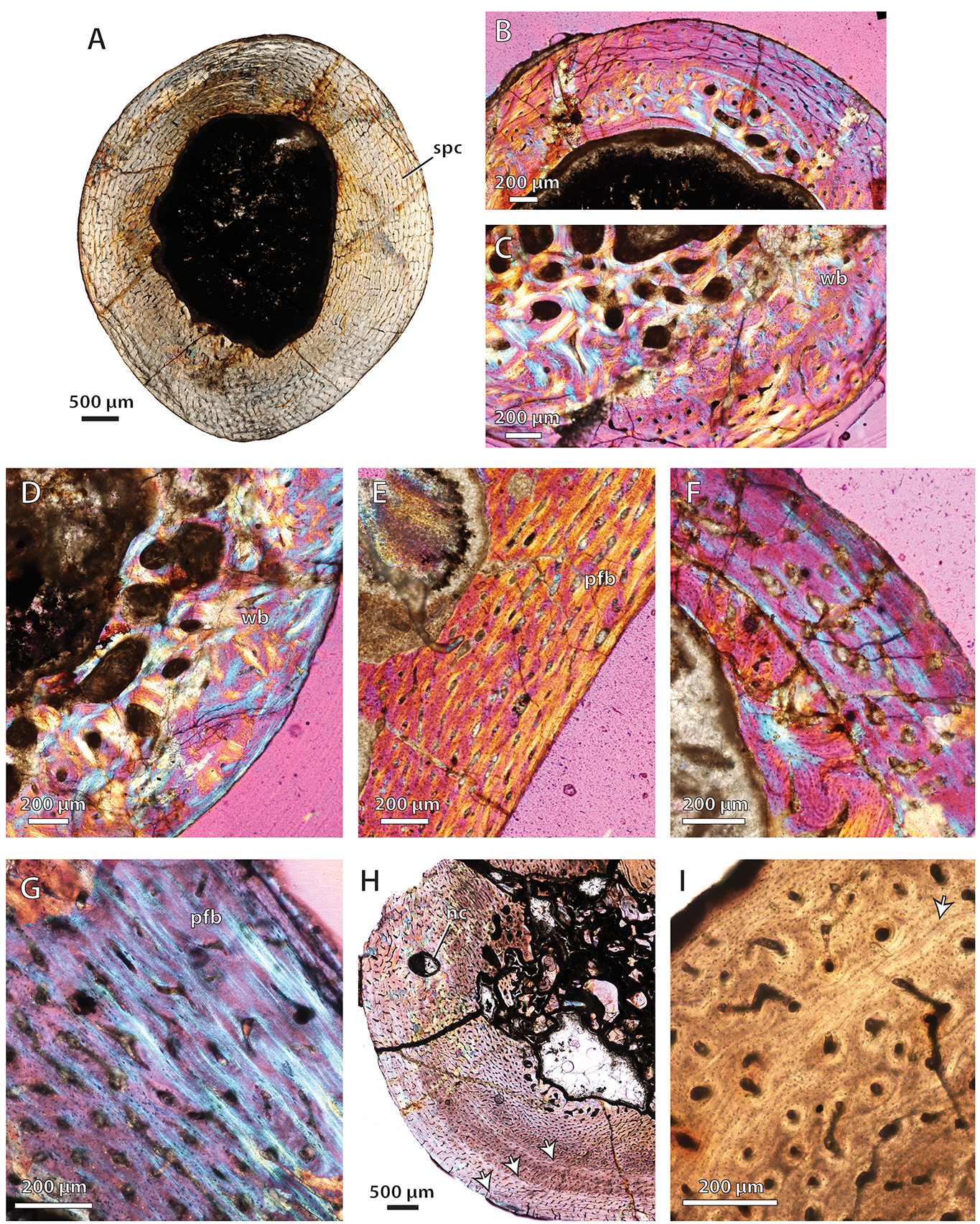

Supplement: Supplemental Information 8 — (A) SAM-PK-K8659, humerus midshaft cross-section viewed at low magnification (normal polarized light). (B) SAM-PK-K8659, radius midshaft cortex and inner cancellous bony scaffold (crossed-nicols with wave plate). (C) SAM-PK-K8659, tibia midshaft cortex and inner cancellous bony scaffold (crossed-nicols with wave plate). (D) SAM-PK-K8659, femur midshaft showing inner cancellous bone and outer bone compacta with a woven-fibered matrix (crossed-nicols with wave plate). (E) SAM-PK-K10423, femur distal shaft cortex (crossed-nicols with wave plate). (F) SAM-PK-K10423, fibula proximal shaft cortex (crossed-nicols with wave plate). (G) BP/1/75, humerus midshaft cortex (crossed-nicols with wave plate). (H) BP/1/4092, midshaft cross-section of large humerus viewed at low magnification (crossed-nicols with wave plate). (I) BP/1/4092, radius close-up showing growth mark (annulus) in outer cortex (non-polarized light). Arrows denote growth marks. Abbreviations: nc, nutrient canal; pfb, parallel-fibered bone; spc, subplexiform canals; wb, woven-fibered bone. [file peerj-02-325-s008.png]

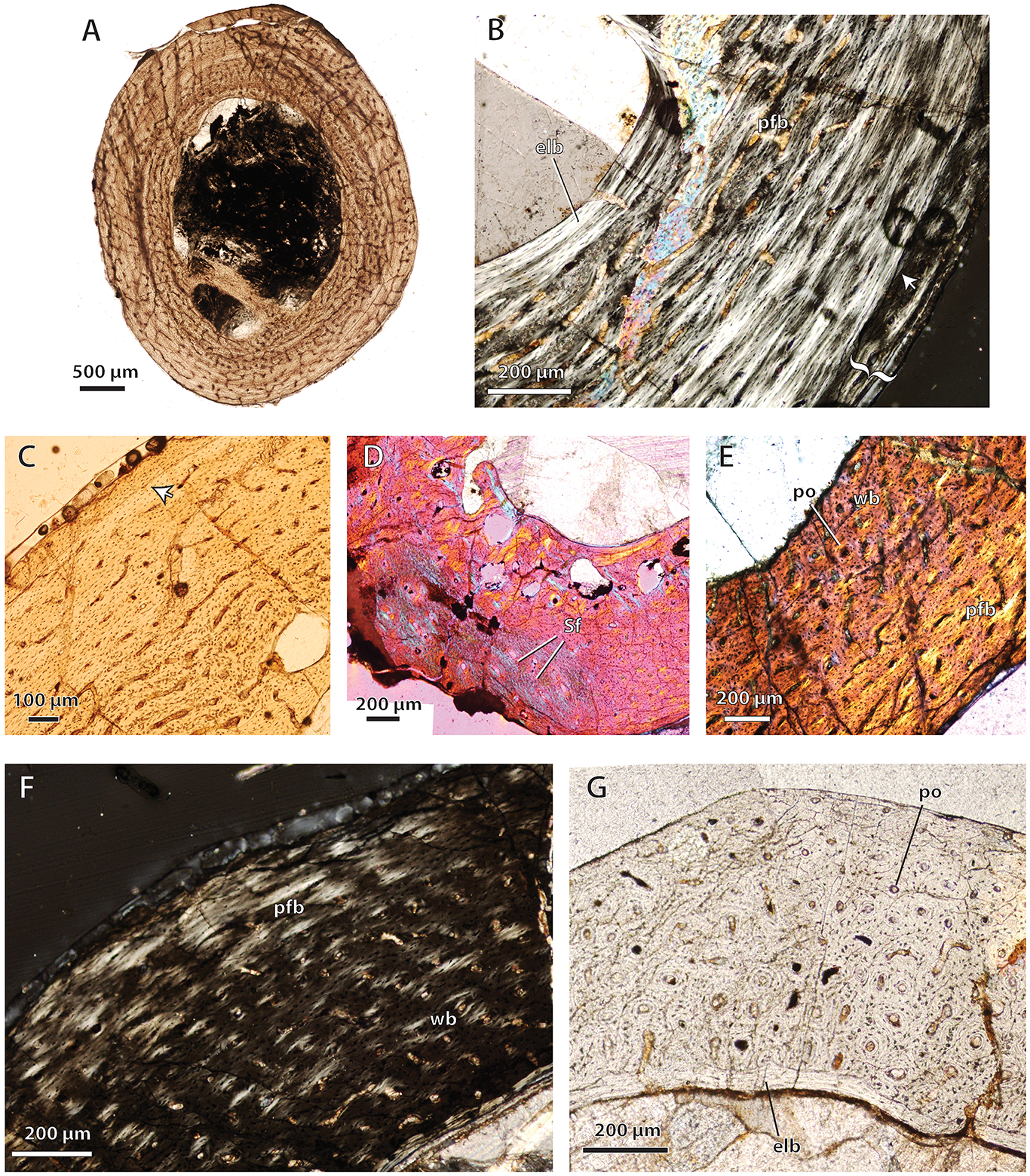

Supplement: Supplemental Information 9 — (A) NMQR 3745, humerus midshaft cross-sectional profile viewed at low magnification (non-polarized light). (B) UCMP 78396, humerus midshaft cortex (normal polarized light at maximum extinction). Bracket denotes outer zone of parallel-fibered and lamellar bone. (C) UCMP 78396, humerus midshaft cortex showing outer line of arrested growth (demarcated by cement line at arrow) (non-polarized light). (D) UCMP 78395, radius midshaft cortex and perimedullary region viewed at low magnification (crossed-nicols with wave plate). (E) UCMP 78396, femur midshaft cortex and perimedullary region showing woven- and parallel-fibered bone (crossed-nicols with wave plate). (F) UCMP 78396, femur midshaft cortex close-up (normal polarized light at maximum extinction). (G) UCMP 78396, fibula midshaft cortical bone packed with longitudinal primary osteons (non-polarized light). Arrows denote growth marks. Abbreviations: elb, endosteal lamellar bone; pfb, parallel-fibered bone; po, primary osteon; Sf, Sharpey’s fibers; wb, woven-fibered bone. [file peerj-02-325-s009.png]

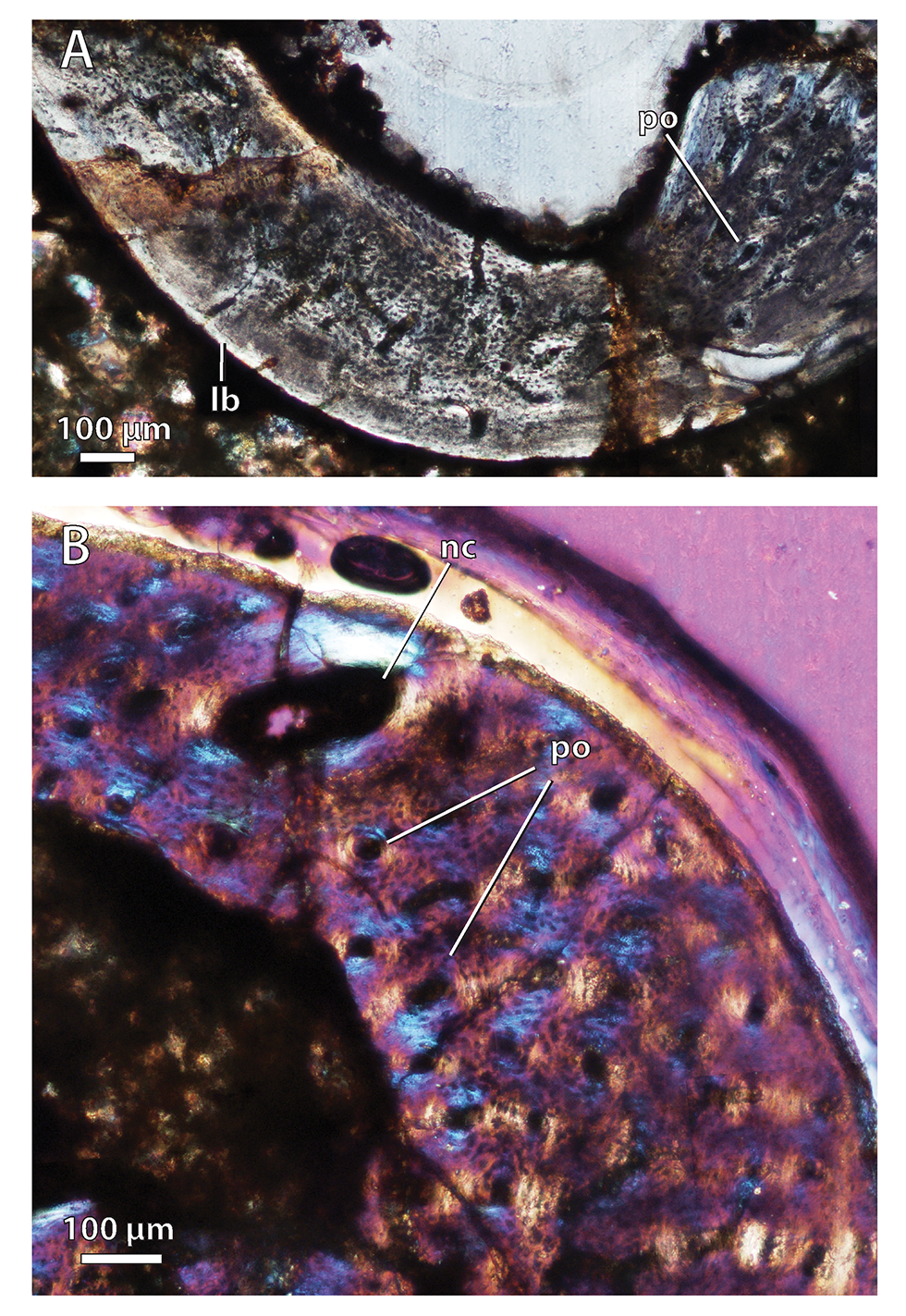

Supplement: Supplemental Information 10 — (A) Humerus midshaft cross-section showing fibrolamellar bone deposition followed by a thin collar of lamellar bone in the subperiosteal region (normal polarized light at maximum extinction). (B) Humerus midshaft cortex close-up showing longitudinal primary osteons and a large nutrient canal within a woven-fibered bone matrix (crossed-nicols with wave plate). Abbreviations: lb, lamellar bone; nc, nutrient canal; po, primary osteon. [file peerj-02-325-s010.png]

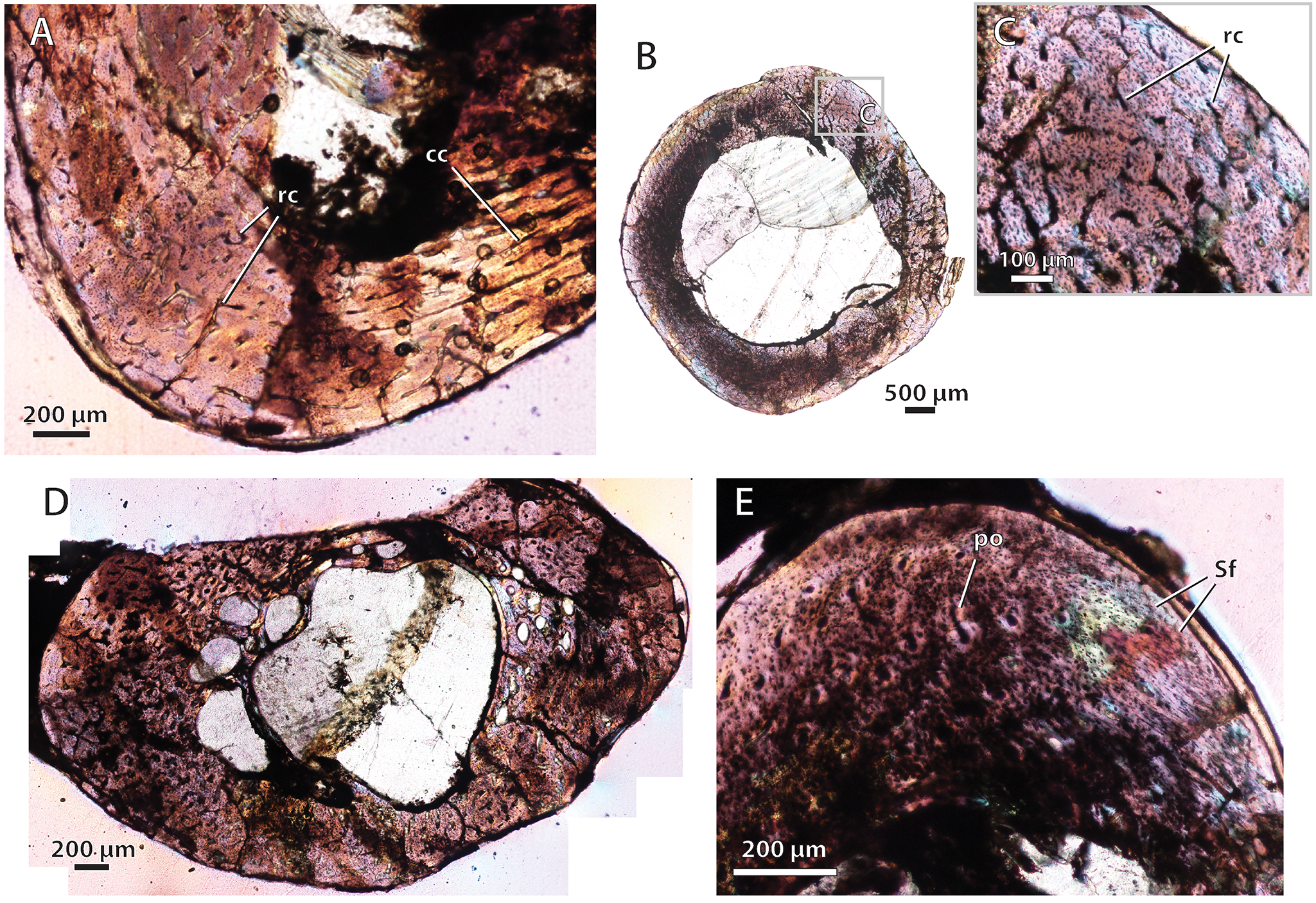

Supplement: Supplemental Information 11 — (A) NMQR 3605, humerus midshaft cross-section viewed at low magnification (crossed-nicols with wave plate). Note local histovariation in the arrangements of vascular canals. (B) NMQR 3605, femur midshaft cross-sectional profile viewed at low magnification (crossed-nicols with wave plate). (C) Close-up of ‘B’ showing abundant reticular canals in cortex and lack of growth marks. (D) NMQR 3605, tibia midshaft cross-sectional profile viewed at low magnification (crossed-nicols with wave plate). (E) NMQR 3605, fibula midshaft cortex (crossed-nicols with wave plate). Abbreviations: cc, circular canal; rc, reticular canal; po, primary osteon; Sf, Sharpey’s fibers. [file peerj-02-325-s011.png]
